# DNA polymerase iota promotes EMT and metastasis of esophageal squamous cell carcinoma by interacting with USP7 to stabilize HIF-1α

**DOI:** 10.1038/s41419-024-06552-6

**Published:** 2024-02-24

**Authors:** Aidi Gao, Mingxia Zhang, Shuang qi Zhu, Shitao Zou, Hengrui Chen, Xiaoqin Li, Chao He, Liangsu Zhou, Yan Mei, Weiqun Ding, Jundong Zhou, Yue Zhou, Yuandong Cao

**Affiliations:** 1 Suzhou Cancer Center Core Laboratory, The Affiliated Suzhou Hospital of Nanjing Medical University, Suzhou, Jiangsu P.R. China; 2 Department of Radiation Oncology, the First Affiliated Hospital of Anhui Medical University, Hefei, P.R. China; 3 Department of Pathology, University of Oklahoma Health Science Center, Oklahoma City, OK USA; 4 Department of Thoracic Surgery, First Affiliated Hospital of Nanjing Medical University, Nanjing, P.R. China; 5 Department of Radiation Oncology, The First Affiliated Hospital of Nanjing Medical University, Nanjing, Jiangsu P.R. China

**Keywords:** Oesophageal cancer, Metastasis

## Abstract

Esophageal squamous cell carcinoma (ESCC) is one of the most lethal cancer types, with a low 5-year survival rate of ~20%. Our prior research has suggested that DNA Polymerase iota (Pol ι), a member of Y-family DNA polymerase, plays a crucial role in the invasion and metastasis of ESCC. However, the underlying mechanism is not well understood. In this study, we utilized ChIP-PCR and luciferase reporter assays to investigate the binding of HIF-1α to the promoter of the Pol ι gene. Transwell, wound healing, and mouse models were employed to assess the impact of Pol ι and HIF-1α on the motility of ESCC cells. Co-immunoprecipitation and Western blot were carried out to explore the interaction between Pol ι and HIF-1α, while qRT-PCR and Western blot were conducted to confirm the regulation of Pol ι and HIF-1α on their downstream targets. Our results demonstrate that HIF-1α activates the transcription of the Pol ι gene in ESCC cells under hypoxic conditions. Furthermore, the knockdown of Pol ι impeded HIF-1α-induced invasion and metastasis. Additionally, we found that Pol ι regulates the expression of genes involved in epithelial-mesenchymal transition (EMT) and initiates EMT through the stabilization of HIF-1α. Mechanistically, Pol ι maintains the protein stability of HIF-1α by recruiting USP7 to mediate the deubiquitination of HIF-1α, with the residues 446–578 of Pol being crucial for the interaction between Pol ι and USP7. Collectively, our findings unveil a novel feedforward molecular axis of HIF-1α- Pol ι -USP7 in ESCC that contributes to ESCC metastasis. Hence, our results present an attractive target for intervention in ESCC.

## Introduction

Esophageal squamous cell carcinoma (ESCC) is the predominant histological subtype of esophageal cancer in China, with a high incidence rate and poor prognosis, ranking seventh in cancer incidence and sixth in overall cancer mortality rate [1]. Although the incidence and mortality rate are in decline, it is still one of the most lethal cancer types with poor prognosis and high metastatic potential [1–4]. Recent studies have focused on elucidating the molecular mechanisms underlying ESCC progression and metastasis. Among the molecules implicated in ESCC progression, DNA polymerase iota (Pol ι) has emerged as a critical player [5–9]. Pol ι is an essential DNA polymerase that plays a key role in the translesion synthesis (TLS) pathway, primarily functioning in DNA damage repair [10, 11]. Translesion DNA synthesis (TLS) is a critical mechanism for cells to repair DNA damage and preserve genomic stability [12, 13]. For instance, Pol θ initiates the repair of DNA double-strand breaks through theta-mediated end joining by using short homologous DNA sequence homologies [14], and Pol μ mediates the incorrect incorporation of dGTP opposite the templating, resulting in the promutagenic T:G mispair and genomic instability [15]. In vitro studies indicate that Pol ι has the lowest fidelity among all eukaryotic polymerases [16, 17]. Additionally, Pol ι has an unusual catalytic pocket structure and favors Hoogsteen over Watson–Crick pairing [18, 19]. Recent research has shown that the regulation of Pol ι involves posttranslational modifications and protein–protein interactions in cancer cells [19–21]. Our previous research has shown that Pol ι is overexpressed and contributes to the progression, invasion, and metastasis of ESCC [5–9]. Specifically, our findings indicate that the increase in Pol ι prompts the expression of genes related to epithelial-mesenchymal transition (EMT) in ESCC cells, suggesting that Pol ι plays a role in promoting metastasis and invasion in ESCC through EMT induction. Despite these findings, the specific molecular mechanisms driving Pol ι-mediated ESCC progression and metastasis remain unclear.

Similar to other solid tumors, hypoxia is closely linked to the development, advancement, spread, blood vessel formation, the transition of cells from epithelial to mesenchymal (EMT), and sensitivity to radiation of Esophageal Squamous Cell Carcinoma (ESCC) [22]. Hypoxia-inducible factor-1α (HIF-1α) is known to govern the response of cells to hypoxia and has been found to be highly active in ESCC, leading to a poor prognosis, spread, invasion, and resistance to therapy [23–26]. In low-oxygen conditions, HIF-1 connects to the hypoxia response element (HRE) and triggers specific genes, including Snail, Slug, and Pol ι [27–29]. The stability of the HIF-1α protein is meticulously managed through posttranslational modifications, notably ubiquitination, with Von Hippel-Lindau (VHL) E3 ubiquitin ligase as the primary regulating factor for HIF-1α [30]. In normoxic conditions, HIF-1α is ubiquitinated and degraded by proteasomes, but in low-oxygen conditions, it detaches from VHL and rapidly accumulates in cells [31, 32]. Nevertheless, HIF-1α ubiquitination can also be reversed by enzymes such as USP7 [33–35]. Despite the recognition of HIF-1α as a crucial regulator of ESCC, the precise molecular mechanisms governing its regulation in cancer cells remain unclear, and the potential for targeting the ubiquitin degradation of HIF-1α in clinical contexts requires further exploration.

In our study, we discovered that HIF-1α plays a crucial role in the regulation of Pol ι expression, and the interaction between Pol ι and USP7 is crucial for stabilizing HIF-1α in low-oxygen conditions. This process is not dependent on VHL and suggests that there may be other mechanisms at play in the regulation of HIF-1α stability in ESCC. It is also noteworthy that this regulatory system seems to initiate the EMT process, which is known to be closely associated with the spread and advancement of cancer. The identification of the Pol ι -USP7 interaction as a prospective therapeutic target is promising and could open up new avenues for the treatment of ESCC. Overall, this study offers valuable insights into the molecular mechanisms underlying ESCC advancement and spread and may have important clinical implications for the development of targeted therapies for this disease.

## Materials and methods

### Patients and sample collection

The clinical samples of esophageal squamous cell carcinoma (ESCC) were procured following a process of obtaining informed consent from all patients, and the related investigations were approved by the Research Ethics Committee of the Affiliated Suzhou Hospital of Nanjing Medical University. All tissue specimens were promptly snap-frozen upon acquisition and preserved at −80 °C to ensure optimal sample quality. The ESCC patient information for the study cohort is shown in Supplementary Table 1 and Supplementary Table 2.

### Immunohistochemical (IHC) staining

Paraffin sections were processed through dehydration and embedding, with subsequent deparaffinization using xylene. Antigens were restored using heated citrate buffer (pH = 6) and blocked with 5% BSA at room temperature for 30 min. Primary antibodies were incubated overnight at 4 °C, followed by the addition of HRP-conjugated anti-mouse/rabbit secondary antibody, which was incubated for two hours at 37 °C. A DAB Substrate kit was used to develop color (Cwbio, China, Cat# P10100), and slides were then counterstained with hematoxylin and dehydrated before a cover slip was applied. Microscopic observations were conducted on five randomly selected fields from every section at a magnification of 100×. The Pol ι or HIF-1α immunohistochemistry scores are calculated as follows: Pol ι/ HIF-1α immunohistochemistry score = (% of positive tumor cells) × the staining intensity. The patients were assigned into two groups with high (IHC score ≥6) and low (IHC score <6), respectively. The antibodies used in this study were anti-Pol ι (13635-1-AP, Proteintech, 1:200) and anti-HIF-1α (#36169, CST, 1:200). The study was approved by the Research Ethics Committee of the Affiliated Suzhou Hospital of Nanjing Medical University and all donors provided informed consent.

### Chromatin immunoprecipitation (ChIP)

ESCC cells were subjected to formaldehyde cross-linking to stabilize protein–DNA complexes. Cross-linking was terminated by adding glycine and incubating at room temperature. After washing with pre-cooled PBS, cells were lysed with SDS lysis buffer supplemented with PMSF and subjected to ultrasonic treatment to shear chromatin into fragments of 200–1000 bp in size. Following centrifugation, the resulting supernatant was incubated with an anti-HIF-1α (CST,36169) antibody and protein A agarose/Salmon Sperm DNA. The antibody/DNA complexes were collected by centrifugation, washed, and eluted with elution buffer. Cross-links were reversed, and the DNA fragments were purified using a gDNA kit and quantified using a NanoDrop2000 instrument. PCR was performed using primers designed to amplify DNA fragments bound to HIF-1α, specifically the Pol ι-pf1-pr, pf2-pr2, and pf3-pr3 regions. The PCR products were analyzed to determine the binding of HIF-1α to Pol ι.

### Cell lines and cell culture

The human esophageal squamous cell carcinoma (ESCC) cell lines ECA-109, TE-1, TE-10, KYSE-30, KYSE-150, and KYSE-510 were procured from the American Type Culture Collection (ATCC) located in Manassas, Virginia, USA. These cells were maintained in DMEM (Hyclone, SH3024.01) and RPMI-1640 (QIDU BIOPHARMACEUTICAL, QL1202) medium supplemented with antibiotics and 10% fetal bovine serum (FBS, Biological Industries, 04-001-1ACS) at 37 °C in a 5% CO_2_ incubator. To study the effects of hypoxia on ESCC cells, they were cultured under hypoxic conditions (1% O_2_) in a nitrogen incubator.

### Luciferase reporter assay

The promoter sequences of Pol ι, Snail, and Slug were obtained from the UCSC website. After amplifying the promoter sequences, they were cloned into the PGL4.17 vector. The negative control vector, positive control vector (PGL4.51), and PGL4.17- Pol ι/Snail/Slug vectors were transfected into TE-1 or ECA-109 ESCC cells using the Lipofectamine3000 Transfection Kit (Invitrogen, L3000-015). Cells were harvested 24 h later and the luciferase activity in the cell lysate was measured using the Dual-Luciferase Reporter Assay Kit (Promega, E1910) on the LUMINOMETER (Progma) after transfection with promoter plasmids. The relative luciferase activity was calculated as follows: Luc (Firefly luciferase)/R-Luc (Renilla luciferase). Three replicate wells were collected for each group to measure the activity.

### Generation of stable ESCC cell lines

The lentiviral-mediated knockdown of Pol ι was performed in the ECA-109 cells by infecting them with lentivirus containing shNC or shRNAs specific targeting to Pol ι followed by selection with 1 μg/mL puromycin. The efficiency of knockdown was confirmed by quantitative reverse transcription PCR (qRT-PCR) and Western blot analysis. TE-1 cells are infected with the lentivirus containing the coding sequence (CDS) of Pol ι mRNA, followed by puromycin treatment. Then, puromycin-resistant cells were analyzed by qRT-PCR and Western blot. Lentivirus containing Cas9 and Pol ι targeting sgRNAs was purchased from Genepharma (Suzhou, China), and ESCC cells were infected with these lentivirus following puromycin selection to knockout Pol ι.

### In vivo metastasis mouse model

In the tail vein metastasis study, female athymic nude mice aged 6–8 weeks were injected with 1 × 10^6^ ECA-109 cells that stably expressed GFP into their tail veins. Each group consisted of six mice. After 10 days, the mice were imaged using a VIS Spectrum system with Living Image software to monitor lung metastatic colonization. Two weeks later, the mice were euthanized, and their lungs were collected for analysis using hematoxylin and eosin (HE) staining and immunohistochemistry (IHC). For the second batch of tail vein injection, mice were monitored at 3, 4, 5, and 6 weeks. Fluorescence imaging was used (AniView100, China) to observe lung metastases every week following tail vein inoculation. For the abdominal cavity metastatic model, female nude mice were intraperitoneally injected with 1 × 10^6^ ECA-109 cells tagged with GFP and intraperitoneal metastasis was monitored by fluorescent imaging. For the axillary lymph node metastasis model, 1 × 10^6^ ECA-109 cells expressing luciferase were injected into the right armpit of the mice. This study was approved by the Research Ethics Committee of the Affiliated Suzhou Hospital of Nanjing Medical University.

### Western blot analysis and antibodies

Cells were harvested and lysed using M-PER Mammalian Protein Extraction Kit lysis buffer (Thermo Scientific, 78501), supplemented with protease and phosphatase inhibitors. Protein quantification was performed using a BCA assay kit, and equal amounts of proteins were separated by SurePAGE™ precast gels with a linear gradient between 4 and 20% (GenScript, Nanjing, China). The separated proteins were transferred to PVDF membranes (Millipore, Billerica, MA, USA) using the eBlot® L1 protein transfer system (GenScript). The membranes were then blocked with 5% non-fat milk and incubated with primary antibodies overnight at 4 °C. After washing three times, HRP-conjugated anti-mouse or anti-rabbit secondary antibody (MultiSciences) was added and incubated for a specific duration. Band visualization was achieved using an ECL substrate, and images of the protein bands were captured using the Tanon-5200 Chemiluminescent Imaging System (Tanon). The expression of β-Tubulin and GAPDH was used as the loading control.

The following antibodies were used: anti-β-Tubulin (Sigma, A5441, 1:5000), anti-Pol ι (Proteintech, 13635-1-AP, 1:1000), anti-HIF-1α (CST, #36169, 1:1000), anti-USP7 (NOVUS, NB100-513, 1:1000), anti-VHL (SANTA CRUZ,sc-17780, 1:500), anti-E-cadherin (Abcam, ab40772, 1:1000), anti-snail (CST, 3879,1:1000), anti-N-cadherin (CST, 4061, 1:1000), anti-Snail (CST, 3879, 1:1000), anti-Slug (CST, 9585 1:1000), anti-ZEB1 (CST, 3396, 1:1000), anti-ZEB2 (Santa Cruz, sc-271984, 1:500), anti-Twist1 (Abcam, Ab50887, 1:1000), anti-Twist 2 (Abcam Ab66031, 1:1000), anti-ubiquitin antibody (Santa Cruz, sc-8017,1:1000), anti-Flag-M2 (Sigma, F1804, 1:5000), anti-HA (Beyotime, AF5057, 1:1000), and anti-MYC antibody (CST, 2276 S, 1:1000). Using Image J software, the intensity of the bands was quantified.

### RNA extract, reverse transcription, and qRT-PCR

Total RNA extraction was performed from TE-1 and ECA-109 cells using Trizol (Sigma, T9424) following standard protocols. Reverse transcription was carried out using 5 μg RNA and a RevertAid RT Kit (Thermo Scientific, K1621). The real-time PCR analysis for SYBR Green was performed using an ABI 7500 real-time PCR system. The ΔCt values were converted to ΔΔCt values by subtracting the average ΔCt values over the test samples from those of the controls. Primers for β-actin, Pol ι, HIF-1α, Snail, and Slug were as follows:

β-actin: F-CACCATTGGCAATGAGCGGTTCC, R-GTAGTTTCGTGGATGCCACAGG,

Pol ι: F-ACTTTCTGCGGTGACTGTGT, R-TACATGGCTTCCCGCATCTC,

HIF-1α: F-GTAAGAAGGCAGTAACCTTTCATCA,

R-AGGGTAGGCAGAACATTTAGGTTTA,

Snail: F-CCCAATCGGAAGCCTAACTA, R-GGACAGAGTCCCAGATGAGC,

Slug: F- CCTTCCTGGTCAAGAAGCAT, R- CACAGTGATGGGGCTGTATG.

### Cell migration assay

The cell migration assay was conducted in Transwell chambers. Using the following protocol, cells were incubated in hypoxic conditions.TE-1 and ECA-109 cells were incubated at 37 °C with 5% CO_2_ and 1% O_2_ using a low oxygen chamber for 12 h and then collected. About 10^5^ cells were added to transwell chambers (Corning) and were allowed to migrate for 24 h. Migrated cells were stained by Wright-Giemsa (Nanjing JianCheng Technology, D010), photographed, and quantified. Cells of six randomly chosen fields were counted from three independent experiments.

### Cell invasion assay

The cell invasion assay involved seeding TE-1 and ECA-109 cells into Transwell chambers coated with Matrigel (Sigma, 356234). The cells were incubated in hypoxic conditions and then incubated in a serum-free RPMI-1640 medium at 37 °C with 5% CO_2_ for 24 h. The lower chambers were filled with RPMI-1640 medium containing 20% FBS. To visualize the invaded cells, Wright-Giemsa (Nanjing JianCheng Technology, D010) staining was performed prior to imaging the chambers. Counts of cells were then conducted in six randomly selected fields of the chamber to quantify the extent of cell invasion.

### Wound healing assay

About 1 × 10^6^ cells were seeded in each well of a six-well plate and allowed to adhere. Scratch wounds were created using a pipette tip, and this process was repeated three times for each condition. The scratched area was quantitatively analyzed using Image J 1.8.0 software. To determine the percentage of scratch closure, the scratch width was measured and photographed under a microscope every 24 h. Each group of cells was selected randomly for three fields of view, and the migration ratio were calculated. The migration ratio was calculated using the following formula: (the width of the original scratch - the width of the actual scratch)/the width of the original scratch × 100.

### Cell adhesion assay

Hypoxia pretreated TE-1 and ECA-109 cells were collected and resuspended in a serum-free culture medium, and diluted to a concentration of 2 × 10^5^ cells/mL. Then, the cells were added 0.2 mL per well to a 96-well plate that was coated with 0.1% gelatin, and incubated under low-oxygen conditions at 37 °C for ~1 h until most cells adhere to the plate. Next, the supernatant was removed, and the plate was washed three times with PBS to discard non-adherent cells, added with fresh serum-free culture medium. Finally, the cell viability was assessed by using CCK8. Each experiment consisted of six wells and was conducted six times.

### Immunofluorescence

The ESCC cells were digested with trypsin and seeded on cell climbing slices for 24 h then the cells were cultured under low oxygen or high oxygen conditions for another 12 h. Cells were washed and fixed with 4% paraformaldehyde and permeabilized with Triton X-100 (Solarbio, T8200). Then, the cells were blocked for 1 h using 5% BSA (MULTI SCIENCE, A90842), followed by incubation with primary antibodies. The antibodies used for immunofluorescence were shown as follows: anti-Pol ι (Proteintech, 13635-1-AP, 1:400), anti-HIF-1α (CST, #36169, 1:800), anti-E-cadherin (CST, 24E10, 1:800). A Dylight 649 conjugated Goat Anti-rabbit antibody (MULTI SCIENCE, A00423) was used as a secondary antibody. Immunofluorescence was detected using confocal laser scanning microscopy (Zeiss, LSM880). Fluorescence images were taken in five fields of each section at random.

### Co-immunoprecipitation (Co-IP)

The ESCC and HEK 293 T cells were subjected to lysis using M-PER Mammalian Protein Extraction Kit lysis buffer (Thermo Scientific, 78501) for protein extraction. Co-immunoprecipitation (co-IP) of Flag-Pol ι, anti-Myc-USP7, or HA-HIF-1α proteins was performed using anti-Flag, anti-Myc, and anti-HA agarose beads (20 µl) for target precipitation, respectively. The endogenous level of HIF-1α was detected using the anti-HIF-1α antibody (Proteintech, 20960-1-AP, 1:100), while rabbit-purified IgG was employed as a negative control. Immunoprecipitation was carried out using protein G-conjugated magnetic beads (Abcam, ab193262) in accordance with the manufacturer’s instructions. Quantitative analysis of the western blotting was analyzed with Image J.

### GST pulldown assays

To perform GST pulldown assays, GST and GST fusion Pol ι proteins were synthesized in *E. coli* BL21(DE3). GST-tagged Pol ι from BL21 was immobilized on glutathione sepharose beads (GenScript, L00206). GST or GST fusion proteins were mixed with MYC-USP7 and HA-HIF-1α expressed HEK293T cell lysates. The eluted proteins were analyzed by western blot.

### In vivo ubiquitination assays

To perform the ubiquitination assay, a co-transfection of Flag-Pol ι and Myc-USP7 plasmids was carried out in the designated cells for 48 h. Following this, the cells were treated with MG132 at a concentration of 20 μM under ambient hypoxia for a period of 12 h. The levels of HIF-1α ubiquitination were determined by immunoprecipitation (IP) using an anti-HIF-1α antibody, followed by western blot assays utilizing an anti-Ub antibody at a dilution of 1:2000 (Santa Cruz, sc-8017). The seeding, transfection, and MG132 treatment of Pol ι and USP7 siRNA-silenced ESCC cells were described earlier in the text. The ubiquitination assays were performed in triplicate and the Western blot analysis was repeated at least three times.

### HIF-1α mRNA stability assay

The stability of RNA was assessed using actinomycin D assays. TE-1 and ECA-109 cells were incubated with 1% O_2_ for 12 h, actinomycin D (GLPBIO, GC16866) was added in cells and collect samples at relevant time points following Actinomycin D addition and proceed to RNA extraction RT-qPCR was used to assess RNA stability. These quantitative PCR data represent the mean ± SEM of three experiments repeated three times.

### HIF-1α protein stability assay

A total of 1 × 10^6^ ESCC cells were seeded into each well of a 6-well plate and allowed to adhere. Following adhesion, the cells were subjected to hypoxic conditions with 1% O_2_ for 12 h. Subsequently, the cells were treated with 50 µg/ml of CHX under normoxic conditions with 20% O_2_ for an additional 4 h. The harvested proteins were collected at the indicated time points and the protein level of HIF-1α was determined by performing Western blot analysis. The cells were cultured under hypoxic conditions for 12 h to accumulate sufficient HIF-1α protein. Subsequently, the culture conditions were switched to 50 μg/mL of CHX while restoring the oxygen concentration to 20% to initiate the degradation of intracellular HIF-1α protein. The CHX chase assay and Western blot were repeated at least three times.

### Statistical analysis

For cell-based experiments, three independent replicates were performed in technical triplicates and each group was measured in duplicate with three biological replicates (experimental replicates). Three replications of all in vitro experiments were performed and one replication of all animal experiments. Animal experiments were repeated in five animals from each group. Two groups were compared using Student’s *t-*test analyses (paired *t*-test or two-sample *t*-test). Correlation analysis of POL ι and HIF-1α mRNA/protein expression in ESCC tissue with Pearson’s chi-square test. For experiments comparing more than two groups, one-way ANOVA or two-way ANOVA(two-sided) with subsequent Dunnett’s multiple comparisons test was used. The results are presented as mean ± standard deviation (SD). A *p* value less than 0.05 was considered statistically significant. All experiments were conducted in triplicate, and data were collected from at least three independent experiments. The statistical calculations were carried out using the SPSS software package. No randomization or blinding was conducted in this study.

## Results

### Pol ι is transcriptionally activated by HIF-1α in ESCC

**Fig. 1:**
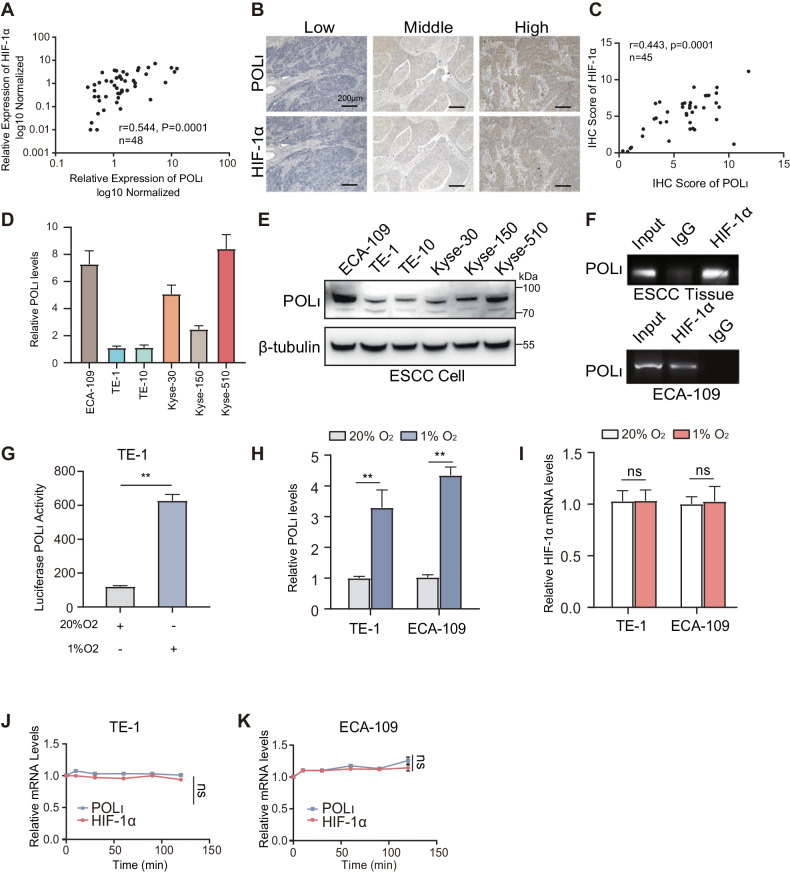
HIF-1α activated the transcription of Pol ι in ESCC. **A** Correlation analysis of POL ι and HIF-1α mRNA expression in Human ESCC tissue (48 cases). **B** Immunohistochemistry staining for POL ι and HIF-1α in ESCC specimens (45 cases, 100×). **C** Correlation analysis of POL ι and HIF-1α protein expression in ESCC tissue with Pearson’s chi-square test. **D** qPCR analysis of Pol ι mRNA expression in ESCC cell lines. **E** Western blot analysis of Pol ι protein expression in ESCC cell lines. **F** Chromatin immunoprecipitation was conducted to assess the binding of HIF-1α to POL ι promoter region in ESCC tissues and in ECA-109 cells. **G** Pol ι promoter reporter luciferase activity was measured using the dual-luciferase reporter assay. **H** qPCR analysis of Pol ι mRNA expression in TE-1 and ECA-109 cells under normoxic and hypoxic conditions. **I** qPCR analysis of HIF-1α mRNA expression after exposure to 20% O_2_ or 1%O_2_. **J**, **K** The mRNA stability of Pol ι and HIF-1α in TE-1 and ECA-109 cells was determined under normoxic and hypoxic conditions (*n* = 3, bar, SD). The data were presented as mean ± SD with statistical significance denoted as *P < 0.05; **P < 0.01; and ns, *P* ≥ 0.05.

To investigate the relationship between HIF-1α and Pol ι in ESCC, 48 ESCC tissue samples were collected and examined the mRNA expression levels of Pol ι and HIF-1α using qPCR. As shown in Fig. 1A, the expression of Pol ι and HIF-1α in clinical ESCC specimens is positively correlated (*R* = 0.544). Next, the correlation was further confirmed by performing IHC staining in 45 ESCC tissues, the results also showed a positive correlation between Pol ι and HIF-1α expression levels (Fig. 1B,C, *R* = 0.443). We subsequently assessed the levels of Pol ι mRNA and protein in ESCC cell lines. The qPCR and western blot analysis revealed that Pol ι is significantly upregulated in ECA-109 and Kyse-510 cells, while TE-1 and TE-10 exhibit lower expression levels compared to other ESCC cell lines (Fig. 1D, E). To investigate HIF-1α‘s role in regulating Pol ι transcription in ESCC tissues and cells, a chromatin immunoprecipitation assay was conducted. As expected, HIF-1α was found to bind to the promoter of the Pol ι gene in both ESCC tissues and cells (Fig. 1F). Previous studies have indicated that Pol ι is a direct transcriptional target of HIF-1α. Therefore, we utilized a dual-luciferase reporter assay to examine the promoter activity of Pol ι under hypoxic conditions, in order to assess the direct impact of HIF-1α expression on Pol ι transcription in ESCC cells. Figure 1G demonstrates a significant increase in promoter activity of Pol ι under hypoxic conditions compared to the normoxic control. Additionally, low-oxygen levels led to elevated transcription of the Pol ι gene in ESCC cells, as shown in Fig. 1H, while HIF-1α transcript levels remained unchanged (Fig. 1I). Furthermore, the stability of both Pol ι and HIF-1α mRNA was not affected, as depicted in Fig. 1J, K. These findings suggest that HIF-1α activates the transcription of the Pol ι gene in ESCC, while Pol ι does not impact HIF-1α transcript levels.

### Pol ι contributes to HIF-1α induced ESCC invasion and metastasis in vitro and in vivo

**Fig. 2:**
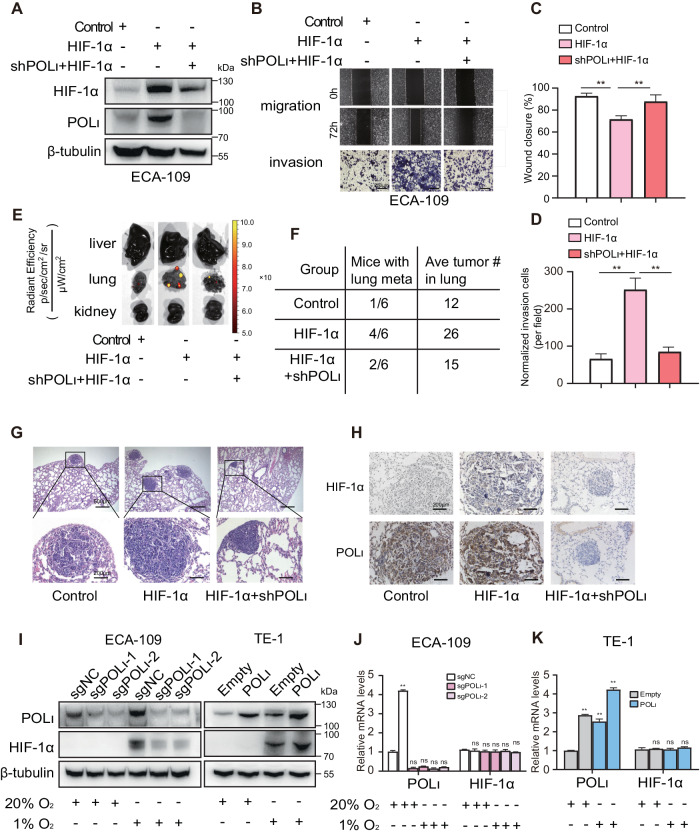
Pol ι contributes to HIF-1α induced ESCC invasion and metastasis in vitro and in vivo. **A** The levels of POL ι and HIF-1α protein were examined by western blot in ECA-109 cells. **B** Wound healing assay and transwell assays were performed to evaluate the migration and invasion ability of ECA-109 cells with or without overexpression of HIF-1α or knockdown of POL ι. **C**, **D** Quantitative analysis of migration and invasion abilities of ECA-109 cells. **E** Fluorescence imaging of live, lung and kidney from mice injected with ECA-109 cells via tail vein. **F** The average number of surface nodules per lung was calculated for each group of mice. **G** Representative images of HE staining of the lung metastatic nodules (40× and 200×). **H** Immunohistochemical analysis of POL ι and HIF-1α expression in the lung tissues from the mice. **I** Western blot analysis of POL ι and HIF-1α protein expression in POL ι overexpressed cells and POL ι knockout cells under hypoxic or normoxic. **J**, **K** qPCR analysis of POL ι and HIF-1α expression in POL ι overexpressed cells and knockout cells under hypoxic and normoxic. The data were presented as mean ± SD with statistical significance denoted as *P < 0.05; **P < 0.01; and ns, *P* ≥ 0.05.

To further investigate the functional relationship between HIF-1α and Pol ι in ESCC cells, ECA-109 cells were stably transfected with the pcDNA3.0-HIF-1α plasmid, which was previously constructed [36]. Then Pol ι was downregulated by shRNA in ECA-109 cells, which was validated by Western blotting (Fig. 2A). Surprisingly, we noticed that the protein level of HIF-1α is reduced after Pol ι downregulation. Since the protein level of HIF-1α is critical to cancer migration and invasion, we next investigated the effects of Pol ι downregulation on ESCC motility (Fig. 2B–D). The results showed that HIF-1α overexpression promoted ESCC cell migration and invasion, while these effects were impeded by Pol ι downregulation.

Lastly, we evaluated the effects of Pol ι knocking down on high-HIF-1α expression ECA-109 cells using a nude mice tail vein metastases model. Bioluminescence imaging demonstrated that HIF-1α promotes the metastasis of ESCC cells in the lung (Fig. 2E). In the Pol ι^-^/HIF-1α^+^ group, the mean number of surface nodules per lung was significantly lower than in the Pol ι^+^/HIF-1α^+^ group (Fig. 2F). Furthermore, HE and IHC staining revealed that the upregulation of HIF-1α led to an increase in the number of metastatic lung nodules, while the silencing of Pol ι reversed the lung metastasis induced by HIF-1α levels in vivo (Fig. 2G, H). To investigate the impact of Pol ι on HIF-1α expression, Pol ι was exogenously expressed in TE-1 cells and knocked out in ECA-109 and Kyse-150 cells using two different guide RNAs. Western blot analysis demonstrated that the deletion of Pol ι in ESCC cells led to a decrease in HIF-1α protein expression, while overexpression of Pol ι resulted in an increase in HIF-1α protein expression under hypoxic conditions (Fig. 2I). Notably, HIF-1α mRNA expression was not influenced by Pol ι (Fig. 2J, K), indicating that Pol ι regulates HIF-1α expression at the post-transcriptional level.

### Pol ι initiates EMT by elevating HIF-1α and its downstream genes under hypoxic environments in ESCC cells

**Fig. 3:**
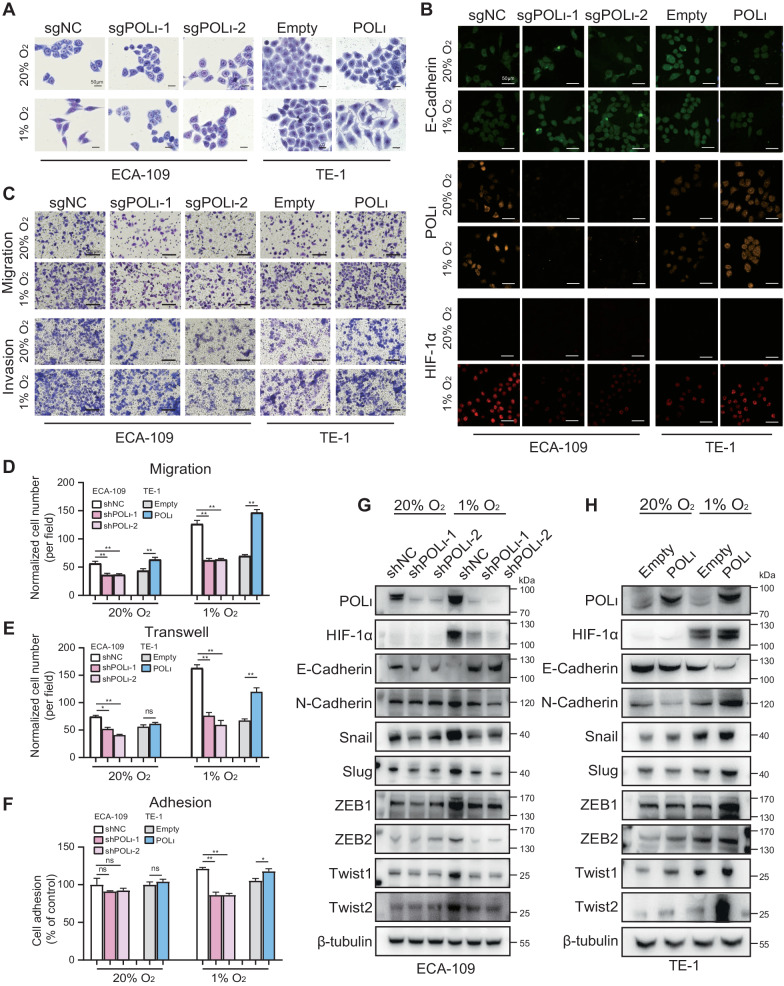
Pol ι initiates EMT by elevating HIF-1α and its downstream genes under hypoxic in ESCC cells. **A** Giemsa staining of ECA-109-sgNC/ECA-109-sgPOLι cells and TE-1-NC/TE-1-POLι cells that were cultured under 20% O_2_ or 1% O_2_ conditions for 12 h (magnification: 400×). **B** Immunofluorescence staining for E-Cadherin, POL ι, and HIF-1α expression in ESCC cells under normoxic and hypoxic. **C** Transwell assay was performed to evaluate the cell migration and invasion ability. **D**, **E** The statistical results of transwell assays. **F** The adhesive ability of the ESCC cells was assessed by an adhesion assay. The data were presented as mean ± SD with statistical significance denoted as *P < 0.05; **P < 0.01; and ns, *P* ≥ 0.05. **G**, **H** EMT-related protein markers were analyzed using Western blot in ECA-109 cells and TE-1 cells after exposure to normoxic or hypoxic for 12 h.

Given that hypoxia activates HIF-1α to induce epithelial-mesenchymal transition (EMT) in cancer cells, we subsequently investigated cellular morphology and performed immunofluorescence to assess the localization of E-cadherin, in order to determine the potential involvement of Pol ι in this process. Pol ι high-expressing cells displayed morphological changes from an epithelial-like phenotype to a loosely connected, elongated, mesenchymal phenotype, while the silence of Pol ι were not driven to undergo EMT processes by hypoxia (Fig. 3A and Fig. S1A, S1B). As depicted in Fig. 3B and Fig. S1C, following a 12-hour exposure to 1% O_2_, immunofluorescence analysis revealed that the suppression of Pol ι led to a decrease in HIF-1α expression and an increase in E-cadherin levels in ECA-109 cells. Conversely, the overexpression of Pol ι markedly elevated HIF-1α expression and facilitated the reduction of E-cadherin. Additionally, the depletion of Pol ι inhibited the migration, invasion, and motility of ECA-109 cells under hypoxic conditions, while the overexpression of Pol ι significantly increased TE-1 cell invasion and migration under both normoxic and hypoxic conditions (Fig. 3C–F). Considering the regulatory effect of Pol ι on HIF-1α expression in ESCC cells, we hypothesized that downstream genes of HIF-1α might also be impacted. Immunoblotting results demonstrated that hypoxia induced the accumulation of HIF-1α, but the knockdown of Pol ι reduced HIF-1α levels in ECA-109 cells. Simultaneously, enhanced expression of E-cadherin and decreased expression of EMT markers such as N-Cadherin, Snail/Slug, ZEB1/2, and Twist1/2 were observed in Pol ι downregulated cells compared to the control cells (Fig. 3G). Furthermore, overexpression of ectopic Pol ι enhanced HIF-1α levels and the expression of its downstream genes in TE-1 cells under hypoxic conditions (Fig. 3H). Additionally, a decrease in HIF-1α protein levels was observed in a dose-dependent manner following the downregulation of Pol ι (Fig. S1D). To understand the mechanism behind this observation, we investigated the activation of Snail and Slug, two transcription factors that are regulated by HIF-1α expression under hypoxia and promote EMT in cancer cells. It was found that knocking down Pol ι inhibited the promoter activities and transcription levels of Snail and Slug, while Pol ι overexpression increased their promoter activities and transcription levels under hypoxic conditions, as demonstrated by qPCR analysis and luciferase reporter assay (Fig. S1E,F).

### Pol ι inhibits the degradation of HIF-1α through the USP7-mediated deubiquitylation

**Fig. 4:**
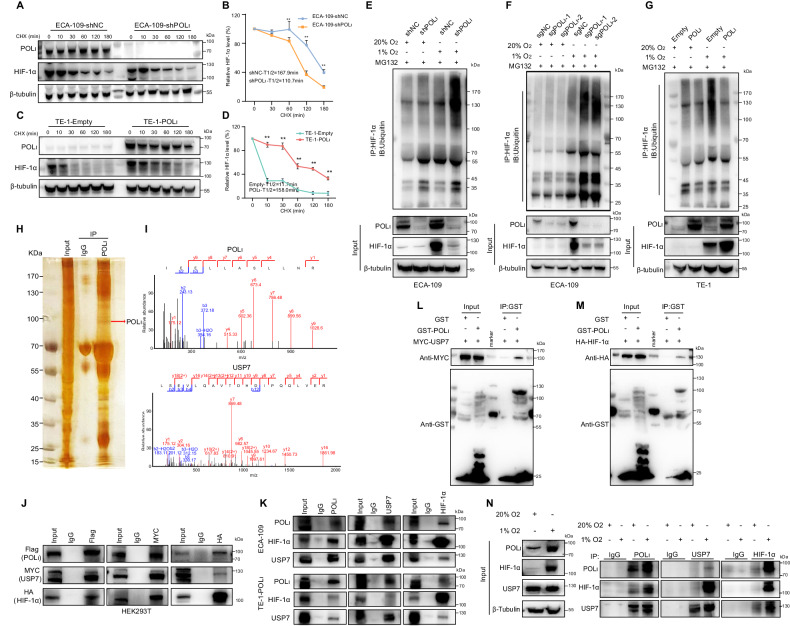
Pol ι inhibits the degradation of HIF-1α through the USP7-mediated deubiquitylation. **A** CHX treatment and western blot assay were conducted to measure HIF-1α protein stability in ECA-109 cells. Cells were incubated with 1% O_2_ for 12 h and then incubated in the presence of CHX for 0, 10, 30, 60,120, or 180 min. **B** Protein half-life curves were made based on the data. Data were presented as mean ± SD (*n* = 3). **C** A CHX chase assay was performed to analyze the half-life of the HIF-1α protein in TE-1 cells. **D** Protein half-life curves were made based on the data. Data were presented as mean ± SD (*n* = 3). **E**, **F** HIF-1α ubiquitination was determined by Western blot with anti-ubiquitin antibody in ECA-109 cells after treatment with 1% O_2_ for 12 h. **G** HIF-1α ubiquitination was determined by western blot with anti-ubiquitin antibody in TE-1 cells after treatment with 1% O_2_ for 12 h. **H** Silver staining was conducted to exhibit POL ι interacted proteins. **I** Mass spectrum analysis of peptides from POL ι and USP7 protein. **J** IP-western blot was conducted to determine the interaction between POL ι, USP7, and HIF-1α in HEK293T cells transfected with Flag- POL ι, Myc-USP7, and HA-HIF-1α. **K** Co-immunoprecipitation of endogenous HIF-1α, POL ι, and USP7 in TE-1 and ECA-109 cells. **L**, **M** GST pulldown assay was performed to determine the interaction between POL ι, USP7, and HIF-1α. **N** Co-immunoprecipitation (co-IP) of endogenous HIF-1α, POL ι, and USP7 in 20% O_2_ or 1% O_2_-exposeded TE-1-NC/TE-1-POL ι and ECA-109-shNC/ECA-109-shPOL ι cells.

The pVHL pathway has been identified as a primary route for the ubiquitination and degradation of HIF-1α. However, the expression of Pol ι did not impact the levels of VHL in ESCC cells (Fig. S1G). Additionally, we did not observe a direct interaction between Pol ι and pVHL in ESCC cells (Fig. S1H). Furthermore, the overexpression of Pol ι did not affect the binding of VHL to HIF-1α (Fig. S1I). These findings suggest the existence of other indirect mechanisms through which Pol ι controls HIF-1α expression in ESCC cells. To investigate whether Pol ι affects the protein stability of HIF-1α, we examined the half-life of HIF-1α protein by treating ECA-109 and TE-1 cells with the protein synthesis inhibitor cycloheximide (CHX) for various time points. Knockdown of Pol ι resulted in a decreased half-life of HIF-1α protein, indicating that Pol ι stabilizes HIF-1α (Fig. 4A, B). Conversely, overexpression of Pol ι increased the half-life of the HIF-1α protein (Fig. 4C, D). Given that the half-life of HIF-1α is intricately regulated through the ubiquitylation-proteasome system, we hypothesize that Pol ι may negatively regulate HIF-1α ubiquitination. The ubiquitination assay using the proteasome inhibitor MG132 revealed that HIF-1α ubiquitination increased in Pol ι downregulated cells, suggesting that Pol ι negatively regulates HIF-1α ubiquitination (Fig. 4E, F). In contrast, overexpressed Pol ι decreased HIF-1α ubiquitination in TE-1 cells under both normoxia and hypoxia (Fig. 4G). These findings indicate that Pol ι modulates HIF-1α stability by regulating its ubiquitination. Notably, it has been reported that USP7 has been demonstrated to interact with Pol ι and modulate the stability of HIF-1α stability [33–35]. To substantiate this hypothesis, an SDS-PAGE was conducted to separate bound proteins, which were subsequently silver-stained (Fig. 4H) and analyzed using mass spectrometry. The MS findings revealed the interaction between Pol ι and USP7 (Fig. 4I). Furthermore, Co-IP results confirmed the presence of Pol ι, USP7, and HIF-1α in the same protein complex in HEK293T cells (Fig. 4J). Similar results were observed in ESCC cells (Fig. 4K). An additional GST pulldown assay indicated in vitro binding between GST-tagged Pol ι and USP7/HIF-1α (Fig. 4L, M). We then examined the interaction among Pol ι, USP7, and HIF-1α under normoxic and hypoxic conditions, respectively. The IP results showed that 1% O_2_ enhances the binding capacity of HIF-1α to both Pol ι and USP7 (Fig. 4N). These findings suggest that Pol ι regulates the stability of HIF-1α by interacting with USP7.

### Pol ι enhances the binding of USP7 to HIF-1α for its deubiquitination via residues 446–578 within the C-terminal of Pol ι

**Fig. 5:**
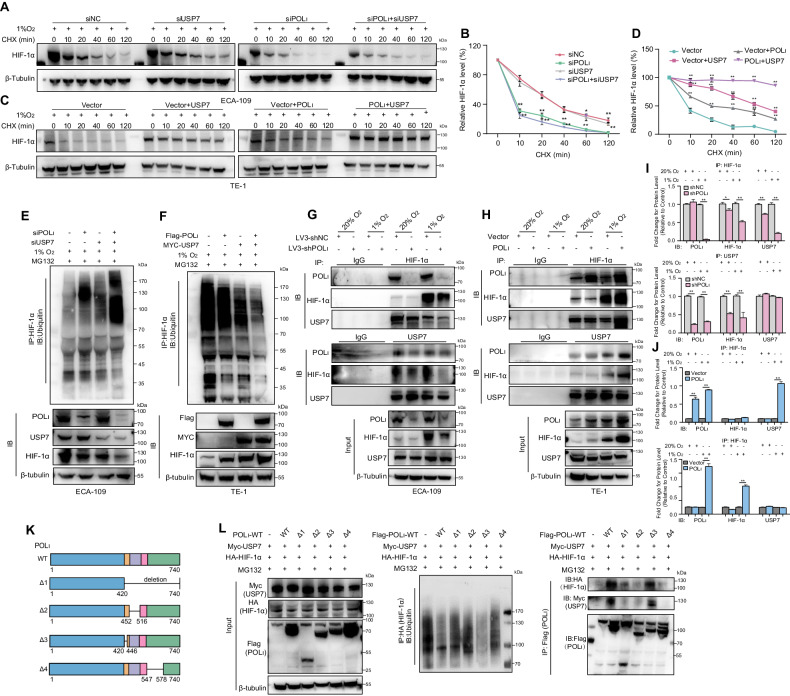
Pol ι enhances the binding of USP7 to HIF-1α for its deubiquitination via residues 446–578aa within the C-terminal of Pol ι. **A** CHX and western blot assays were used to measure the HIF-1α protein stability in POL ι and USP7 knockdown ECA-109 cells. **B** Protein half-life curves were made based on the data in ECA-109 cells. **C** CHX and western blot assays were used to measure HIF-1α protein stability in POL ι and USP7 overexpressed TE-1 cells. **D** Protein half-life curves were made based on the data in TE-1 cells. Data were presented as mean ± SD (*n* = 3). **E** Western blot analysis of the HIF-1α-immunoprecipitated lysates with the anti-ubiquitin antibody in ECA-109 cells. **F** Western blot analysis of the HIF-1α-immunoprecipitated lysates with the anti-ubiquitin antibody in TE-1 cells. **G**, **H** Co-IP assay was performed to determine the binding ability of USP7, POL ι, and HIF-1α in POL ι/USP7 knockdown or overexpression ESCC cells incubated under 1 or 20% oxygen. **I**, **J** Quantitative analysis of the co-IP interaction data. **K** Construction of POL ι wild-type (WT) and deletion mutants with Flag-tag. **L** In vivo ubiquitination assay and Co-IP were conducted to detect HIF-1α ubiquitination and the binding of POLι mutants to USP7 in HEK293T cells.

In light of the potential for hypoxia to enhance the formation of the Pol ι -USP7- HIF-1α complex, which could influence HIF-1α stability, our initial focus was on confirming the role of USP7 in regulating HIF-1α expression in ESCC cells. As anticipated, USP7 knockdown resulted in decreased protein levels of HIF-1α and its downstream targets (Fig. S2A–C). Furthermore, USP7 knockdown led to increased HIF-1α ubiquitination (Fig. S2D). Thus, we investigated whether co-knockdown of Pol ι and USP7 would impede HIF-1α protein stability in ESCC cells under hypoxic conditions. A cycloheximide (CHX) chase assay revealed that individual or combined silencing of Pol ι and USP7 led to a noticeable decrease in HIF-1α stability. Co-depletion of Pol ι and USP7 significantly reduced the stability of endogenous HIF-1α in ESCC cells (Fig. 5A, B and Fig. S3A). On the contrary, co-upregulation of Pol ι and USP7 extended the half-life of HIF-1α protein (Fig. 5C, D and Fig. S3B). Moreover, our ubiquitination assays showed that simultaneous silencing of Pol ι and USP7 increased the ubiquitination level of HIF-1. The siPol ι- siUSP7 knockdown cells exhibited lower HIF-1α protein levels compared to controls (Fig. 5E). In contrast, overexpression of Pol ι and USP7 led to decreased HIF-1α ubiquitination. Higher HIF-1α protein levels were observed in the Flag-Pol ι^+^/Myc-USP7^+^ transfected cells than in the control cells under hypoxic conditions (Fig. 5F). Furthermore, to confirm the association of USP7 with HIF-1α in differentially expressed ESCC cells, Co-IP of Pol ι and USP7 was examined by Western blot under normoxic or hypoxic conditions, following HIF-1α immunoprecipitation. Compared with shNC controls, Pol ι knockdown weakened the binding of HIF-1α with USP7, while Pol ι overexpression clearly enhanced the interaction between HIF-1α and USP7 under 1% O_2_ (Fig. 5G, H). As shown in Fig. 5I, J, the quantification of associated proteins after IP were consistent with the observed trends.

In order to elucidate the specific Pol ι domain responsible for interaction with USP7 and its role in mediating HIF-1α ubiquitination, we generated various deletion mutants of Pol ι with Flag tags (as depicted in Fig. 5K) and co-transfected them with Myc-USP7 and a HA-HIF-1α vector into HEK293T cells. We then examined whether those aforementioned Pol ι mutants impair Pol ι function in modulating HIF-1α ubiquitination. The functional impact of Pol ι mutants on HIF-1α ubiquitination modulation was investigated, and it was observed that mutants Pol ι-Δ1, 2, and 4 failed to reduce the ubiquitination of HIF-1α. Furthermore, the co-IP results demonstrate that both USP7 and HIF-1α specifically bind Pol ι-Δ3, which is located at residues 446–578 within the C-terminal of Pol ι (Fig. 5L).

### Blocking the specific binding of Pol ι and USP7 inhibits HIF-1α induced EMT in ESCC cells

**Fig. 6:**
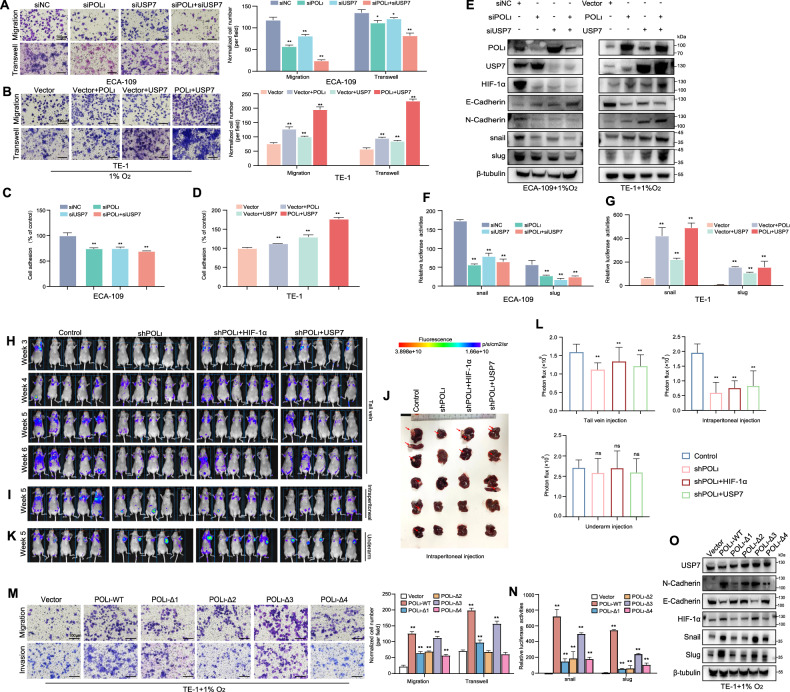
Blocking the specific binding of Pol ι and USP7 inhibits HIF-1α induced EMT in ESCC cells. **A** Images and statistics of the migrated and invaded ECA-109 cells with Pol ι and USP7 knockdown (left panel). The numbers of normalized cell numbers were quantified (right panel). The data (mean ± SD, *n* = 3) are expressed as normalized cell numbers. **P < 0.01. **B** Transwell assay was conducted to evaluate the effects of Pol ι and USP7 co-expression on the migration and invasion of TE-1 cells. The numbers of normalized cell numbers were quantified (right panel). Representative images and statistical analysis. The data (mean ± S.D, *n* = 3) are expressed as normalized cell numbers. **P < 0.01. **C**, **D** Adhesion assays to examine adhesion capacity of Pol ι and USP7 knockdown/overexpression ECA-109 and TE-1 cells. **E** The EMT-related protein levels of Pol ι, USP7, HIF-1α, and EMT-related markers in ECA-109 and TE-1 cells were detected by western blot. **F**, **G** Promoter activities of the Snail and Slug genes in ECA-109 and TE-1 cells was measured using a Dual-Luciferase Reporter Assay. The data are presented as mean ± SD with statistical significance denoted as *P < 0.05; **P < 0.01; and ns, *P* ≥ 0.05. **H** Live animal imaging of mice at various time points after tail vein injection of ECA-109 cells expressing GFP. **I** In vivo imaging of mice after intraperitoneal inoculation of ECA-109 cells expressing GFP. **J** Mice showed liver and spleen metastases after intraperitoneal injection with ECA-109 cells expressing luciferase. **K** In vivo imaging of mice injected with ECA-109 cells expressing GFP via the left armpit. **L** Results of quantitative and statistical analyses of the bioluminescence signal intensities in the NC, shPOLι, shPOLι + HIF-1α, and shPOLι + USP7 groups. **M** Representative image of the transwell assay to evaluate the migration and invasion ability of TE-1 cells expressing different POLι mutants (right panel). The quantitative and statistical analyses are based on normalized cells per field (left panel). **N** Luciferase reporter assays were used to measure the snail and slug promoter activities in TE-1 cells transfected with empty vector, POLι-WT, and POLι-Δ1–4 plasmids. (*n* = 3, bar, SD). **O** EMT-associated protein expression in POLι mutants was analyzed through a western blot assay.

To validate the hypothesis that Pol ι and USP7 are essential for HIF-1α-mediated ESCC cell metastasis, transwell, and adhesion assays were conducted. As depicted in Fig. 6A, C, knockdown of Pol ι and USP7 inhibited the migration, invasion, and motility of ECA-109 cells under low-oxygen conditions, while cells overexpressing Pol ι/USP7 showed significantly increased migration and invasion abilities compared to the control (Fig. 6B, D). Consistently, co-knockdown of Pol ι and USP7 led to a significant reduction in N-Cadherin, Snail, and Slug levels and an increase in E-Cadherin expression. Co-transfection with Pol ι or USP7 alone, or both Pol ι and USP7 further decreased E-Cadherin and increased N-Cadherin, Snail, and Slug expression (Fig. 6E). Additionally, a positive correlation was observed between the protein levels of Snail, Slug and Pol ι/USP7 in ESCC cells. Similar patterns were also detected in the promoter activities (Fig. 6F, G).

Consistent with our above findings, nude mice injected with ESCC cells intravenously demonstrated reduced metastatic ability in the Pol ι knockdown group, while the overexpression of HIF-1α and USP7 partially reversed the decreased migratory ability caused by Pol ι knockdown (Fig. 6H and Fig. S3C). Besides lung metastasis resulting from tail vein injection, we also observed tumor formation in the liver and spleen after intraperitoneal injection of ESCC cells, particularly in the group with high expression of Pol ι. On the contrary, mice in the Pol ι knockdown group showed a significant decrease of metastasis nodules in the liver (Fig. 6I, J and Fig. S3D). However, the restoration of HIF-1α and USP7 in ESCC cells partially restored their migratory ability. But no similar effect on metastasis was observed in the group of mice injected with tumor cells near the axillary lymph nodes (Fig. 6K and Fig. S3E), as indicated by the in vivo fluorescence signals (Fig. 6L). Furthermore, the results of the transwell assay in the Pol ι-Δ1,2 and 4 mutant cells indicated that the migration and invasion abilities of these mutant cells were significantly hindered (Fig. 6M). Moreover, the luciferase activities in Pol ι-Δ1, 2, and 4 expressed cells were nearly 40% decreased in comparison to cells expressing the wild-type Pol ι, while the luciferase activity in Pol ι-Δ3 expressed cells was more than 80% retained (Fig. 6N). In line with the aforementioned findings, only the Pol ι-Δ3 cells showed changes in the levels of E-Cadherin, N-Cadherin, Snail, Slug, and SOX2 as well as Pol ι-WT cells (Fig. 6O). These findings indicate that the C-terminal region of Pol ι, specifically residues 446–578, is crucial for its role in regulating HIF-1α and promoting ESCC EMT.

## Discussion

Despite the advancements in diagnostic and therapeutic methods, ESCC patients still face a high risk of early metastasis and therapeutic resistance [1–3], which is often associated with hypoxia in ESCC cells [37, 38]. Therefore, it is crucial to identify the underlying mechanisms of hypoxia-induced ESCC metastasis. We have established a connection in this research between the expression of Pol ι and HIF-1α in both ESCC tissue samples and cell lines. We have shown that they play interdependent roles in promoting the EMT process and aiding in invasion and metastasis, both in in vitro and in vivo. Through our mechanistic studies, we have uncovered a new feedback loop of HIF-1α‐Pol ι regulation in ESCC, underscoring the significance of targeting this pathway for the development of effective therapeutic approaches for ESCC.

Similar to Ito et al. [29], our findings demonstrate that HIF-1α has the ability to interact directly with the promoter region of the Pol ι gene, which in turn increases its transcriptional activity in ESCC cells. This conclusion is supported by multiple experimental results. Firstly, our chromatin immunoprecipitation assay detected direct binding of HIF-1α to the Pol ι gene promoter, observations consistent with a previous study [29]. Secondly, the luciferase reporter assay demonstrated that the binding of HIF-1α to the Pol ι gene promoter enhances its activity. This finding provides further evidence of HIF-1α’s regulatory role in Pol ι gene transcription. Additionally, qRT-PCR analysis confirmed that the activation of HIF-1α leads to elevated levels of Pol ι transcripts. As we previously reported, the overexpression of Pol ι in ESCC may be regulated by Sp1[5]. This study offers new evidence supporting the involvement of HIF-1α in the overexpression of Pol ι in ESCC, in addition to Sp1. Our findings uncover a novel regulatory mechanism in which HIF-1α interacts with Pol ι in ESCC cells.

On the other hand, the dysregulated expression of Pol ι has been found to influence the expression and activity of HIF-1α, consequently contributing to the initiation of the EMT process in ESCC. Moreover, our study demonstrates that Pol ι enhanced hypoxia-induced expression of EMT markers and promoted the migration and invasion of ESCC cells in a low-oxygen environment. In addition, we validated that the reduction of Pol ι attenuated the metastasis ability of ESCC cells triggered by the overexpression of HIF-1α in vivo. Furthermore, overexpression of USP7 partially reinstated the impaired in vivo metastatic capability of ESCC cells by downregulation of Pol ι. Taken together, these findings indicate that the Pol ι- HIF-1α-USP7 axis can potentiate the metastatic capacity of ESCC cells.

**Fig. 7:**
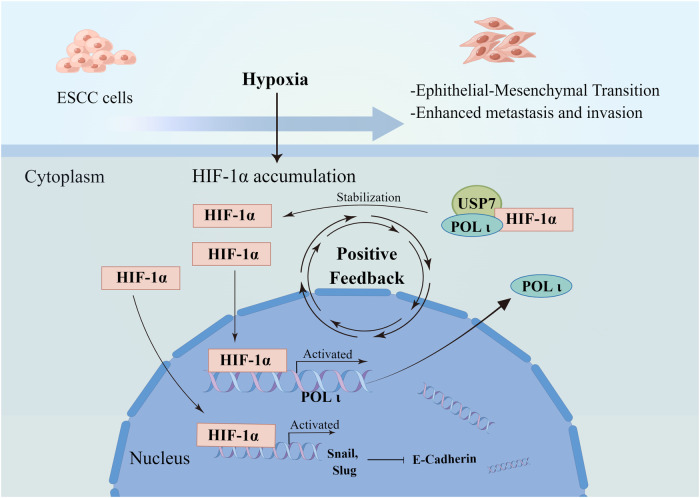
Diagram of the feedforward loop of the HIF-1α-POLι-USP7 regulatory axis. The figure was created using Figdraw.

Mechanistically, Pol ι recruits the deubiquitinase USP7 to enhance its interaction with HIF-1α, and thereby stabilizing the HIF-1α protein. Posttranslational modifications, specifically ubiquitination, play a critical role in the precise regulation of HIF-1α. As a “ubiquitin writer,” VHL modifies HIF-1α with ubiquitin molecules, which subsequently targets it for proteasomal degradation. Despite the well-established role of VHL in HIF-1α ubiquitination and degradation, emerging evidence suggests the existence of a VHL-independent mechanism that contributes to the regulation of HIF-1α, yet its underlying mechanism remains poorly understood [30]. In contrast to “ubiquitin writers” such as VHL, USP7 is classified as a “ubiquitin eraser” or deubiquitinase responsible for the removal of ubiquitin molecules from target proteins. Increasing evidence suggests that USP7 stabilizes HIF-1α through its deubiquitination [33–35]. However, the detailed mechanisms remain unclear. Importantly, our findings demonstrate a significant role of Pol ι in the interactions between USP7 and HIF-1α. According to the report by Nicholas W and Valles GJ, the C-terminal region of Pol ι was found to interact with the TRAF-like and UBL1-2 domains of USP7 [34, 35]. Our findings demonstrated that the residues located between 446 and 578 within the C-terminal region of Pol ι are essential for the deubiquitination, stabilization, and functional activity of HIF-1α and blocking the specific binding of Pol ι and USP7 inhibits HIF-1α induced EMT in ESCC cells (Fig. 7).

## Conclusions

In this study, we demonstrate a reciprocal relationship between HIF-1α and Pol ι, whereby the activation of HIF-1α enhances Pol ι transcription, and alterations in Pol ι expression affect the stability of HIF-1α protein and subsequent progression of ESCC through enhanced binding of HIF-1α with USP7. Our data supports the existence of a novel feedforward loop involving the HIF-1α- Pol ι-USP7 regulatory axis in ESCC cells, which offers a promising potential therapeutic target to address the rapid progression and therapeutic resistance observed in this disease.

### Supplementary information

Supplementary Figure Legends

Supplement Figure 1

Supplement Figure 2

Supplement Figure 3

Supplement Table 1

Supplement Table 2

A reproducibility checklist

Full and uncropped western blots

## Data Availability

The dataset(s) supporting the findings of this study are included within the article. Requests for materials should be addressed to JZ.
