# Supplementary material for: DNA polymerase iota promotes EMT and metastasis of esophageal squamous cell carcinoma by interacting with USP7 to stabilize HIF-1α
Source: Cell Death Dis. 2024 Feb 24;15(2):171. doi: 10.1038/s41419-024-06552-6 (PMC10894303; doi:10.1038/s41419-024-06552-6)
Supplement: Supplementary file 1 — Supplementary Figure Legends [file 41419_2024_6552_MOESM1_ESM.docx]

**Supplementary Figure Legends**

**Fig. S1** (A) Western blot was used to detect the transfection efficiency of the overexpression plasmid and knockout plasmid in TE-1, ECA-109 and Kyse-150. (B) Giemsa staining was performed to analyze the morphology of Kyse-150 cells. (C) Immunofluorescence staining for E-cadherin, POLι and HIF-1α in RESCC cells merged DAPI signals. (D) EMT-related proteins markers were detected using Western blot in shPOLι or siPOLι silencing ESCC cells after 20% O2 or 1% O2 conditions for 12h. (E and F) QT-PCR and luciferase assay to detect Snail and Slug transcription level in ESCC cells with overexpression or knockdown of POLι after exposure to normoxic or hypoxic conditions for 12 hours. (G) Western blot analyzed expression level of VHL in ECA-109-shNC and ECA-109-shPOLι/ TE-1-NC and TE-1-POLι cells. (H) Co-immunoprecipitation of POLι with VHL in ECA-109 and TE-1 cells. (I) Co-immunoprecipitation assay identified interaction between HIF-1α and VHL in TE-1-NC and TE-1-POLι cells.

**Fig. S2** (A, B) QT-PCR and Western blot analysis of mRNA and Protein levels of USP7 in small interfering RNA knockdown ECA-109 and HEK293T cells. (C) Western blot analysis of HIF-1α and EMT-related proteins level in ECA-109 and HEK293T cells. (D) Ubiquitination assay of HIF-1α in ECA-109 and HEK293T cells with USP7 knockdown.

**Fig. S3** (A) CHX assay and Western blot assay measuring POLι , USP7 and HIF-1α protein stability in POL ι and USP7 knockdown ECA-109 cells. (B) CHX assay and Western blot assay measuring POLι , USP7 and HIF-1α protein stability in POL ι and USP7 overexpressed TE-1 cells. (C) (D) Live mouse imaging at indicated time points after injection.

**Supplement Table 1** The detailed ESCC patient cohort information

**Supplement Table 2** The correlation between POL ι expression and ESCC patients’ pathological features.
