# Supplementary figures and images for: DNA polymerase iota promotes EMT and metastasis of esophageal squamous cell carcinoma by interacting with USP7 to stabilize HIF-1α
Source: Cell Death Dis. 2024 Feb 24;15(2):171. doi: 10.1038/s41419-024-06552-6 (PMC10894303; doi:10.1038/s41419-024-06552-6)

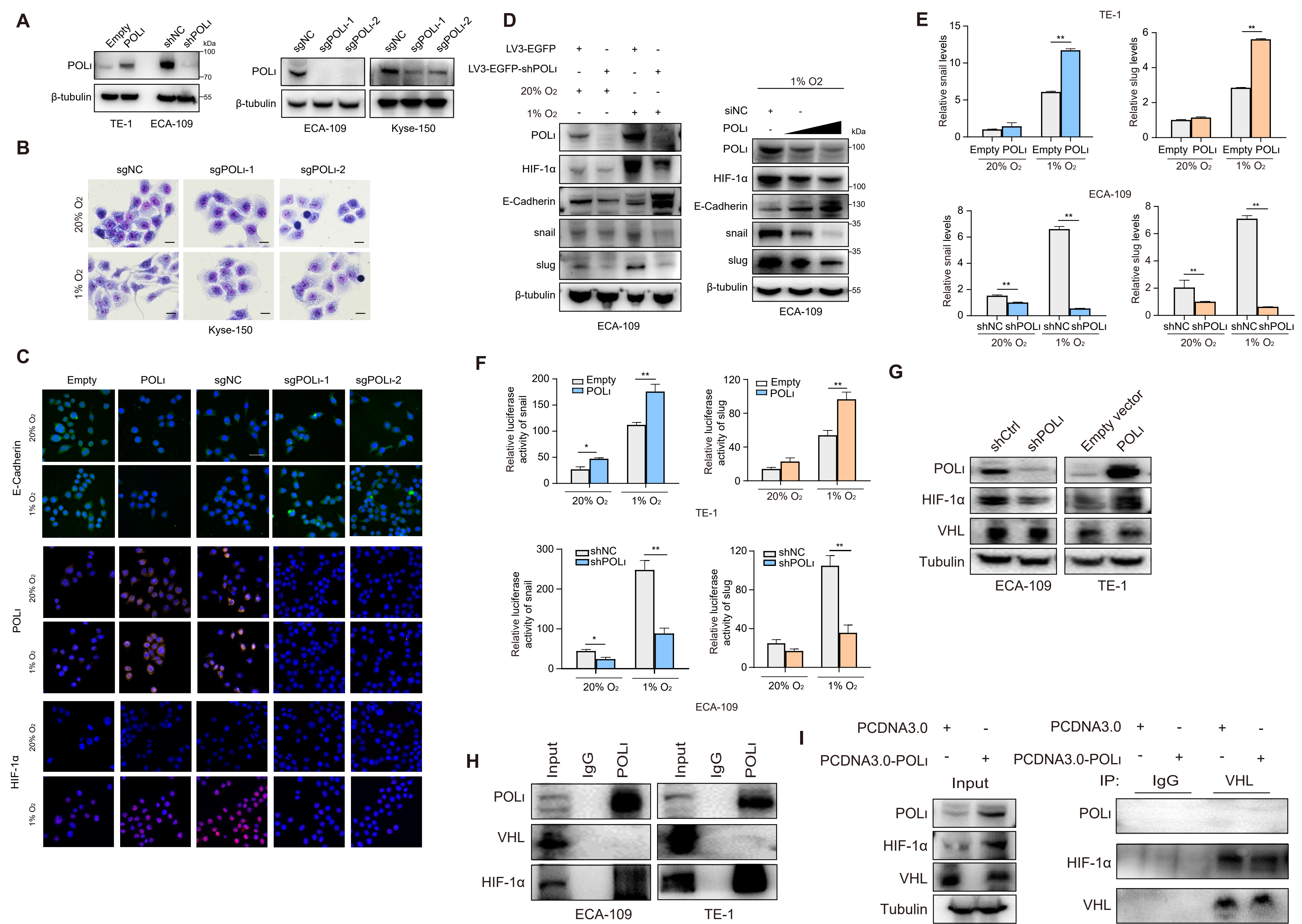

Supplement: Supplementary file 2 — Supplement Figure 1 [file 41419_2024_6552_MOESM2_ESM.pdf]

**A**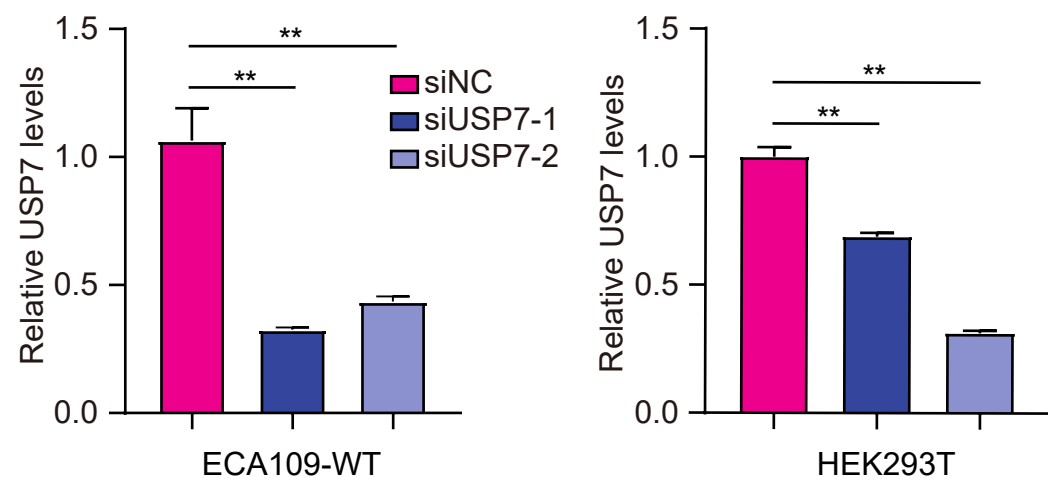**C**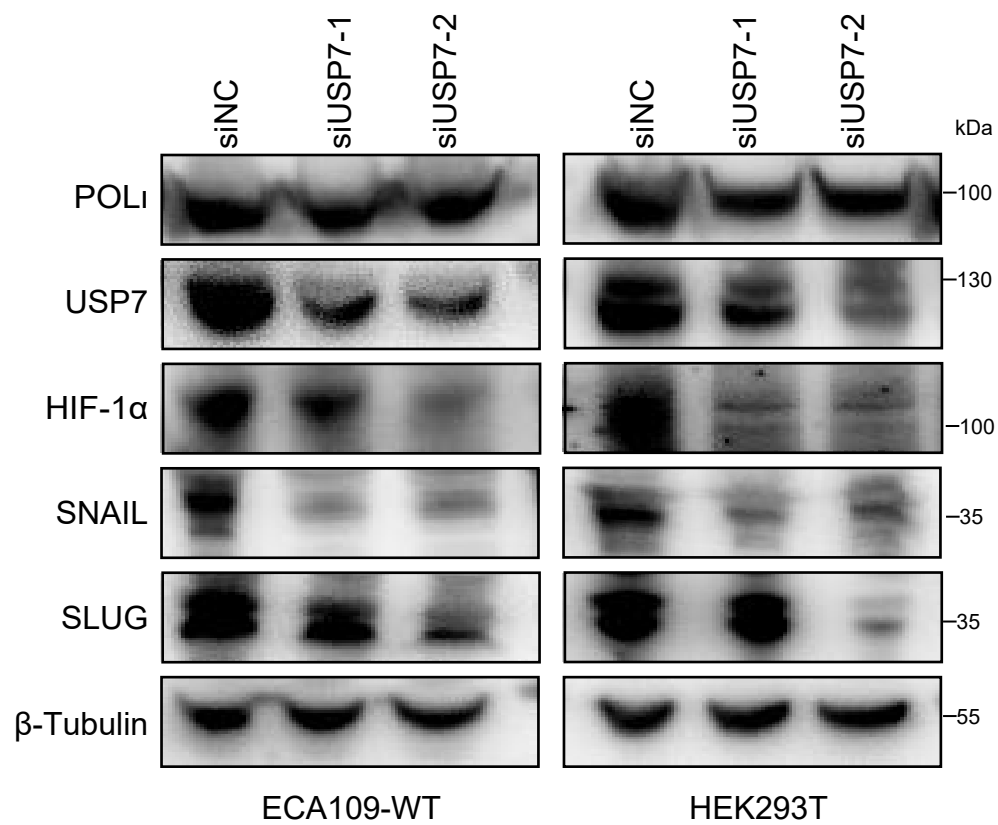**B**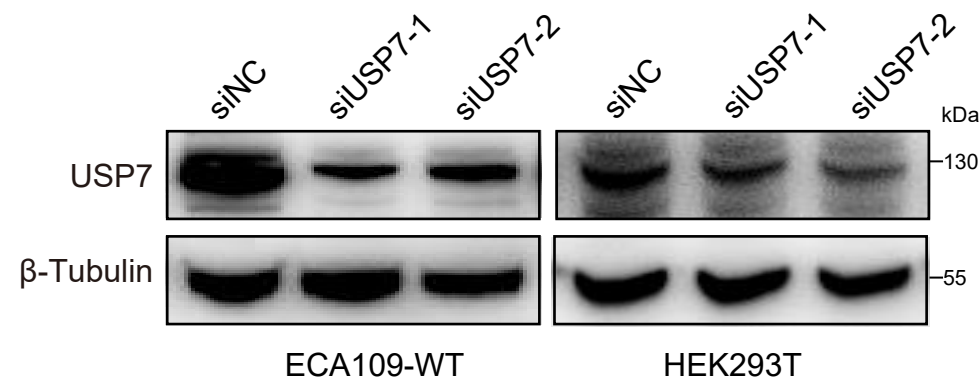**D**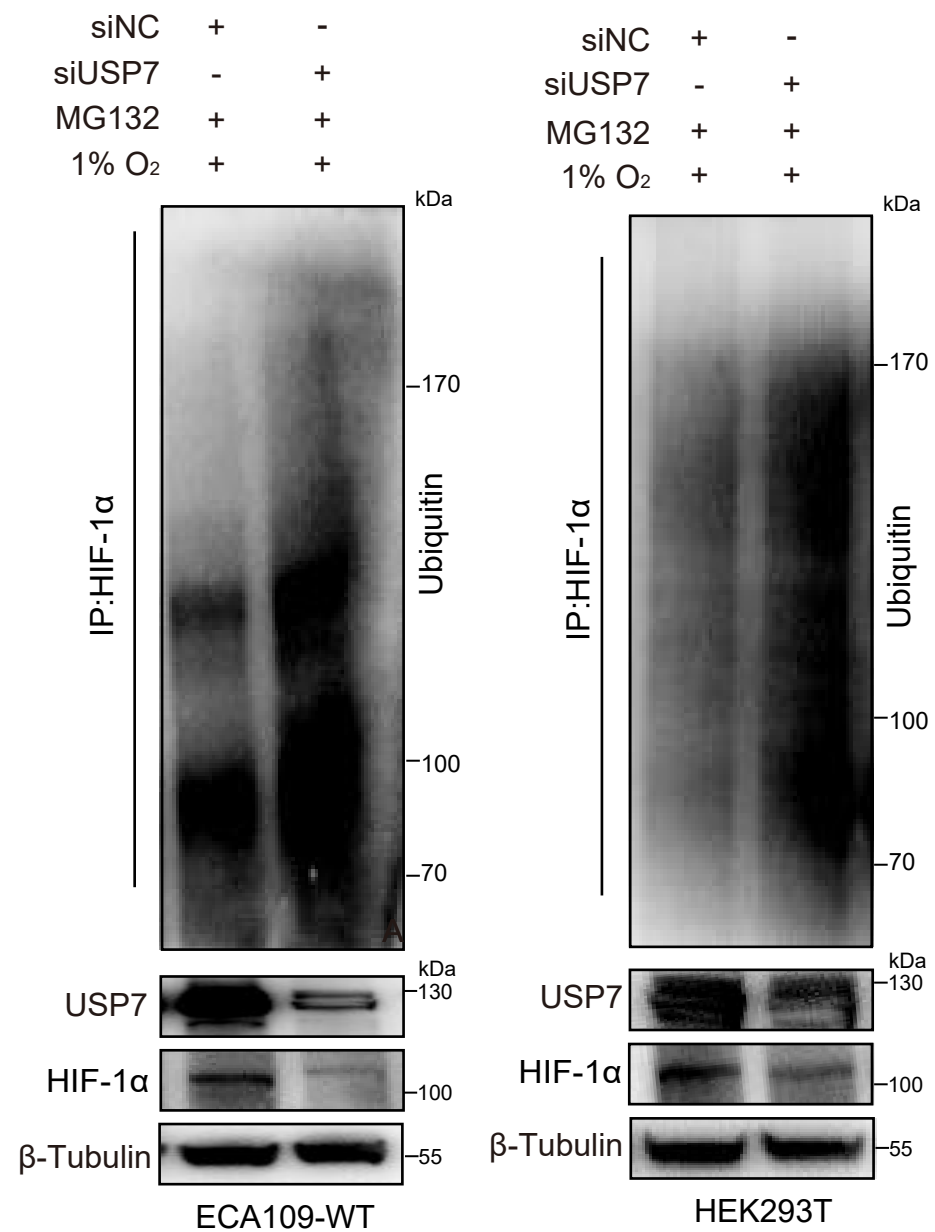

Supplement: Supplementary file 3 — Supplement Figure 2 [file 41419_2024_6552_MOESM3_ESM.pdf]

**A**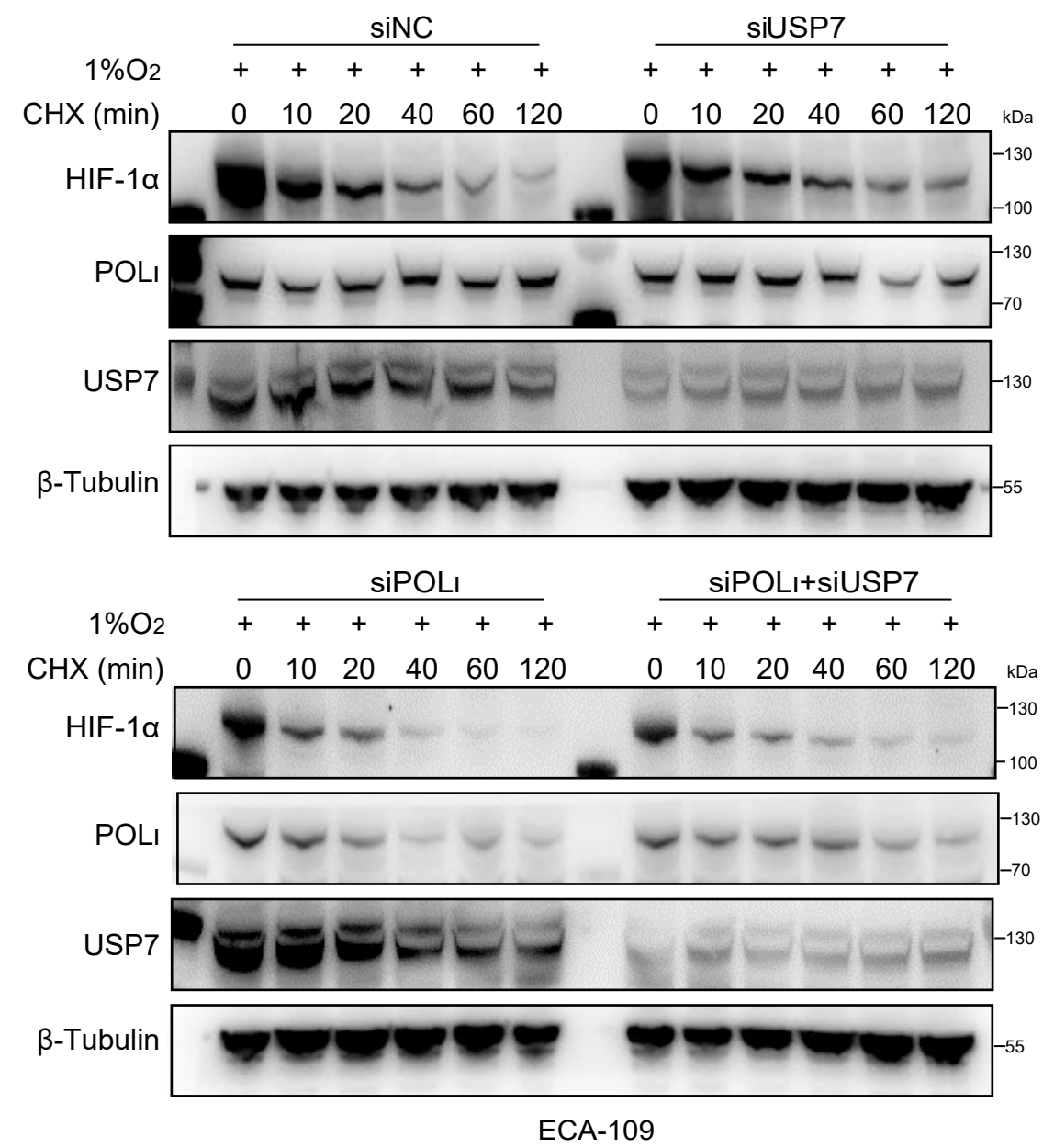**B**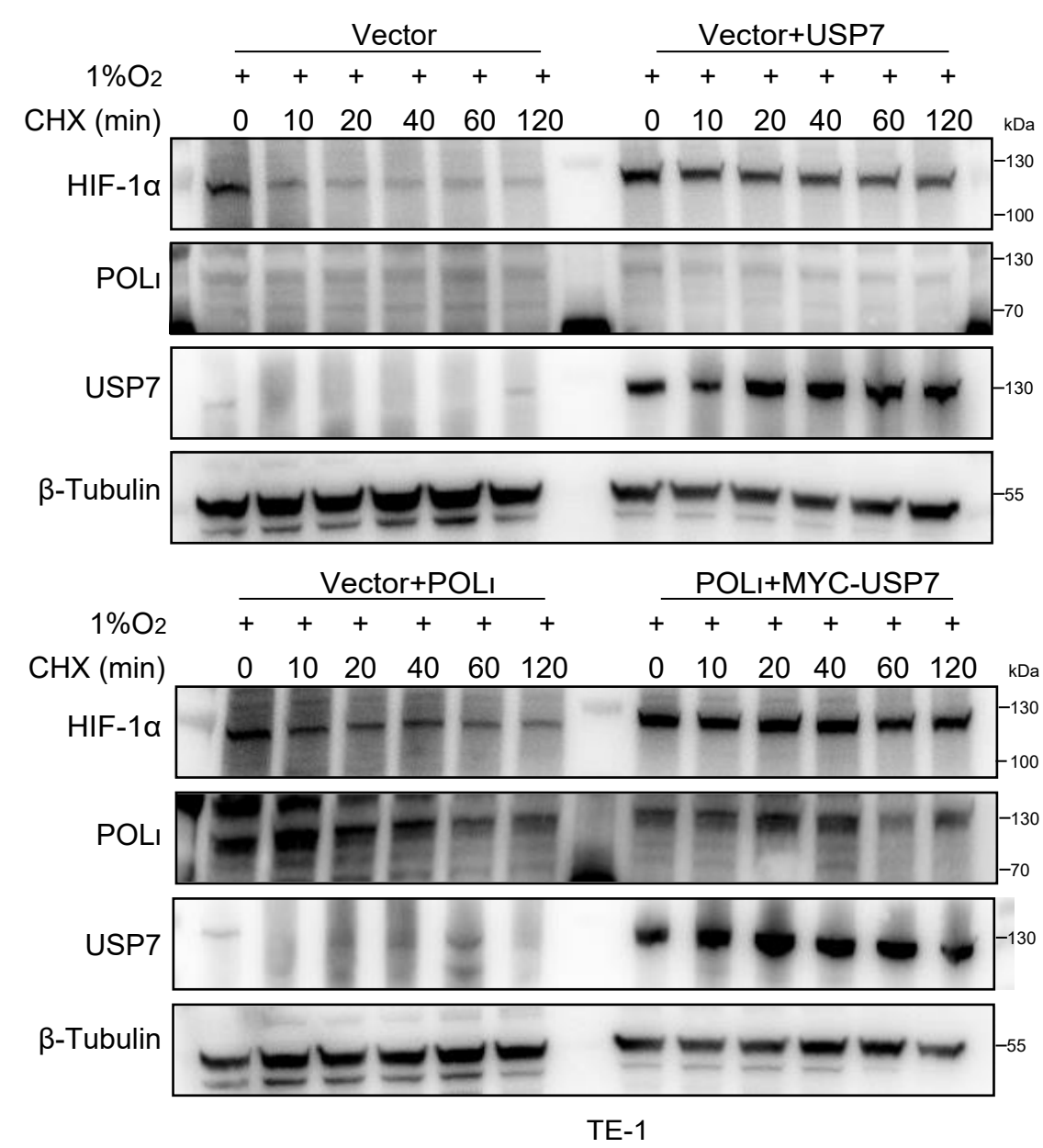**C**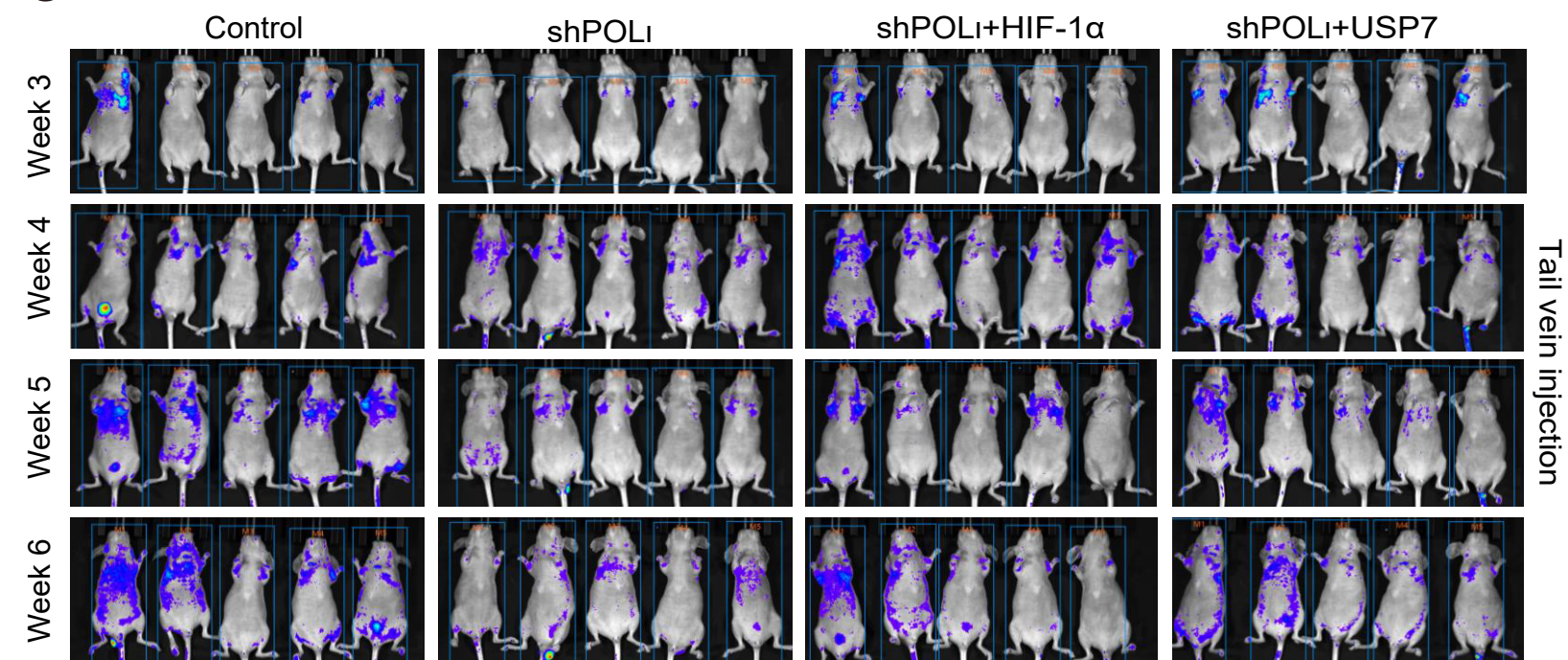**D**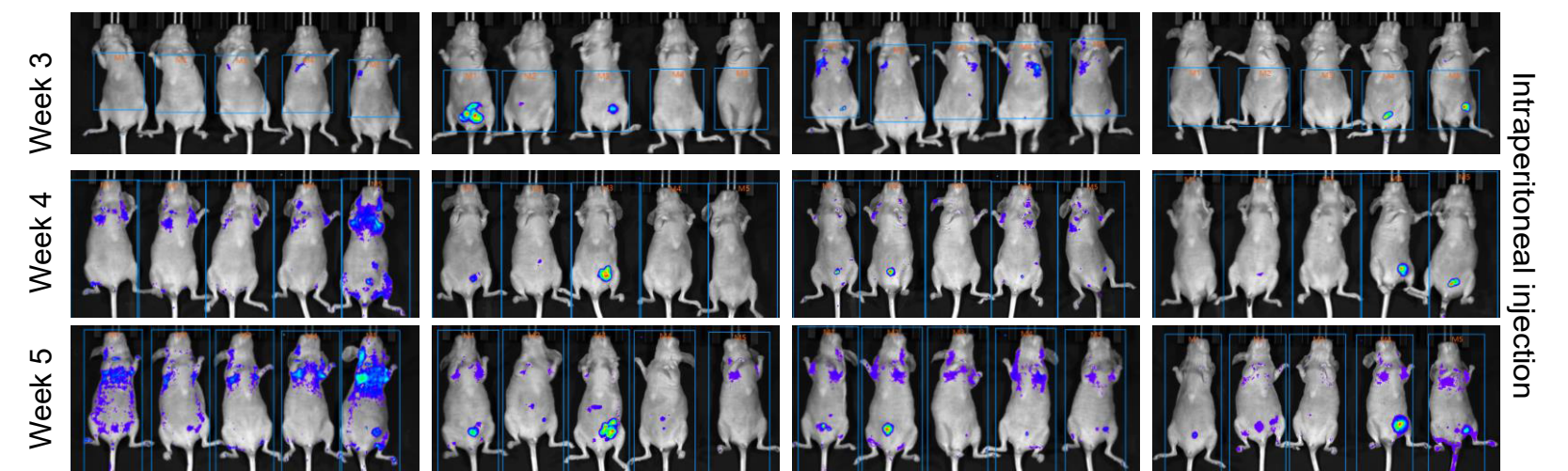**E**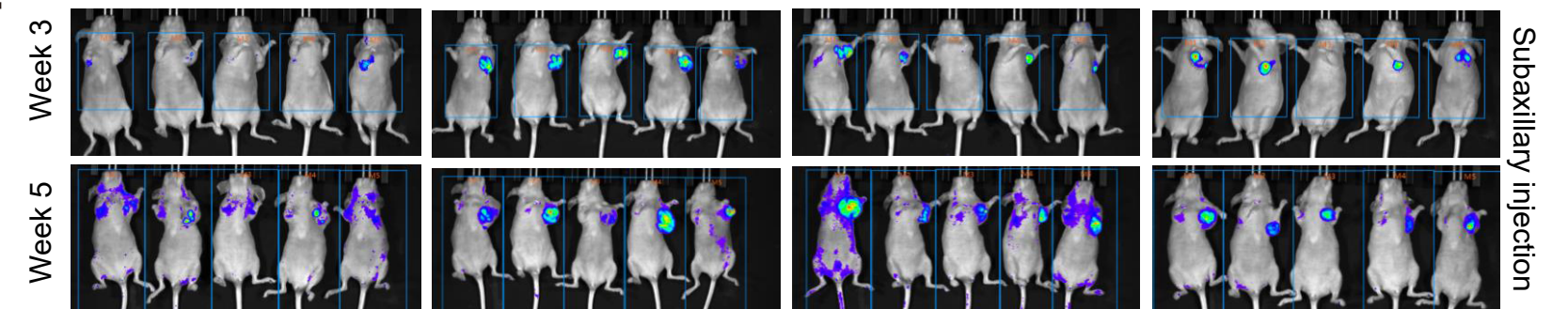

Supplement: Supplementary file 4 — Supplement Figure 3 [file 41419_2024_6552_MOESM4_ESM.pdf]
