# Supplementary material for: DNA polymerase iota promotes EMT and metastasis of esophageal squamous cell carcinoma by interacting with USP7 to stabilize HIF-1α
Source: Cell Death Dis. 2024 Feb 24;15(2):171. doi: 10.1038/s41419-024-06552-6 (PMC10894303; doi:10.1038/s41419-024-06552-6)
Supplement: Supplementary file 6 — Supplement Table 2 [file 41419_2024_6552_MOESM6_ESM.docx]

**Table1. The correlation between Pol ι expression and ESCC patients’ pathological features.**

| **Characteristics** | **Pol ι expression** | | **P value*** |
| --- | --- | --- | --- |
|  | low | high |  |
| **Gender** |  |  | 0.7833 |
| Male | 12 | 16 |  |
| Female | 8 | 9 |  |
| **Age** |  |  | 0.3358 |
| ≤60 | 6 | 11 |  |
| >60 | 14 | 14 |  |
| **T stage** |  |  | 0.0007** |
| T0-T2 | 19 | 12 |  |
| T3-T4 | 1 | 3 |  |
| **N stage** |  |  | 0.0035** |
| N0 | 14 | 8 |  |
| N1  N2  N3 | 5  1  0 | 8  4  5 |  |
| **TNM stage** |  |  | 0.0792 |
| Ⅰ  Ⅱ | 12  6 | 7  11 |  |
| Ⅲ | 2 | 7 |  |
| Pathological grade  Ⅰ  Ⅱ-Ⅲ  Ⅳ | 11  9  0 | 2  20  3 | 0.0015** |

*chi-square test. *P＜0.05
