# Supplementary material for: DNA polymerase iota promotes EMT and metastasis of esophageal squamous cell carcinoma by interacting with USP7 to stabilize HIF-1α
Source: Cell Death Dis. 2024 Feb 24;15(2):171. doi: 10.1038/s41419-024-06552-6 (PMC10894303; doi:10.1038/s41419-024-06552-6)

Figure 1

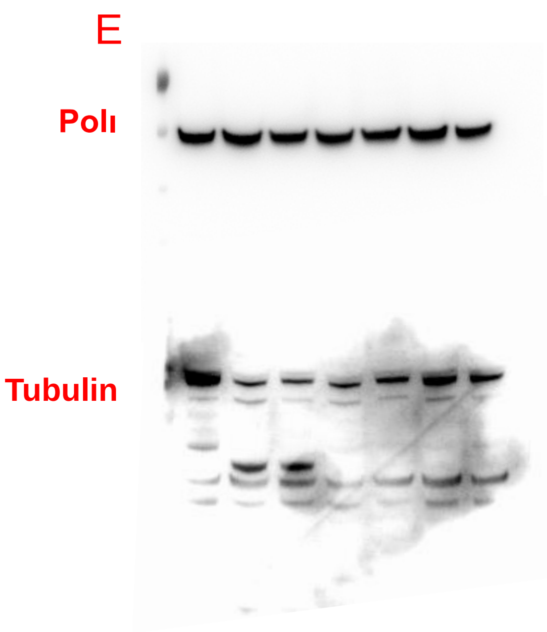

Figure 2

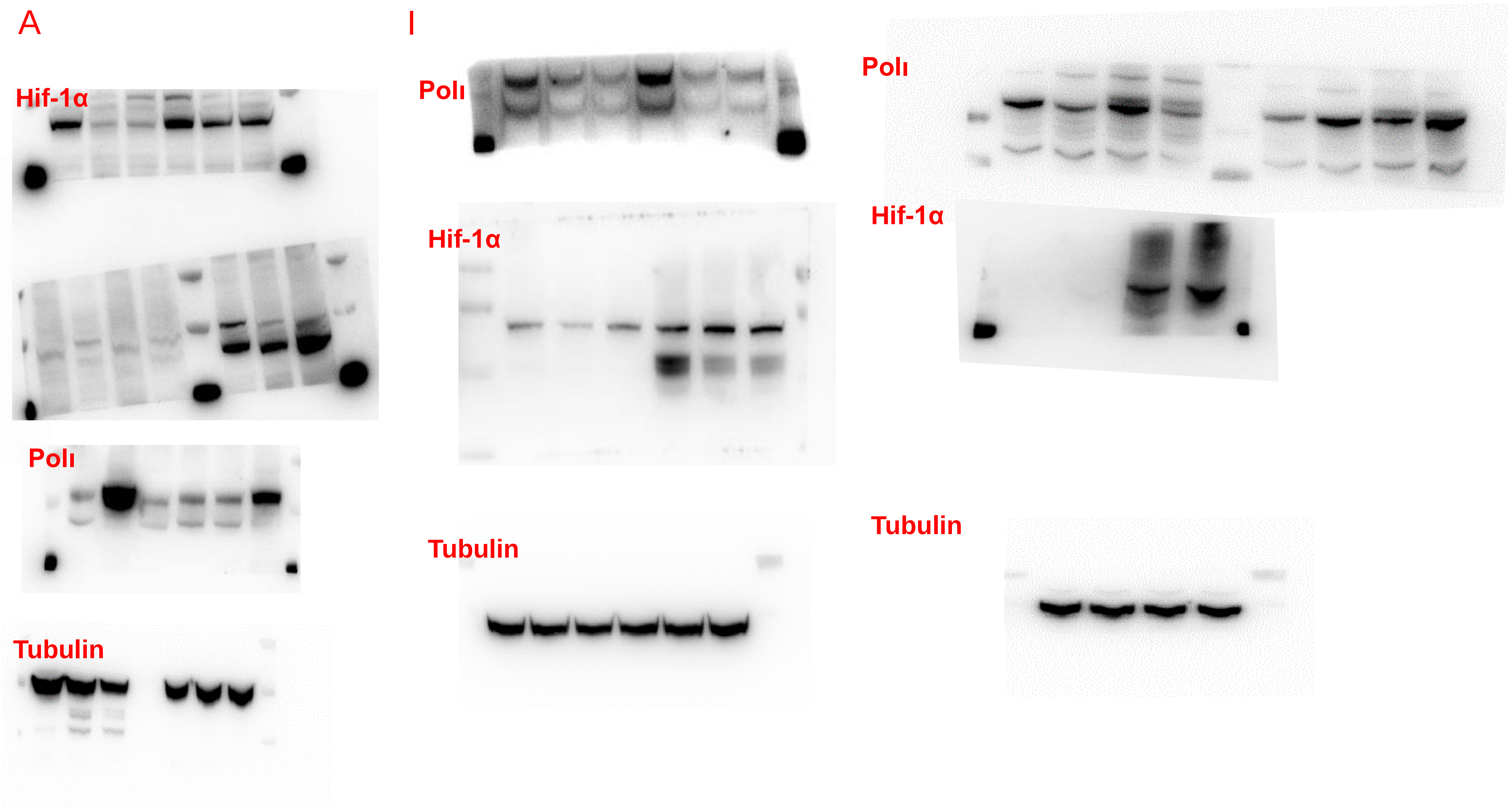

Figure 3G

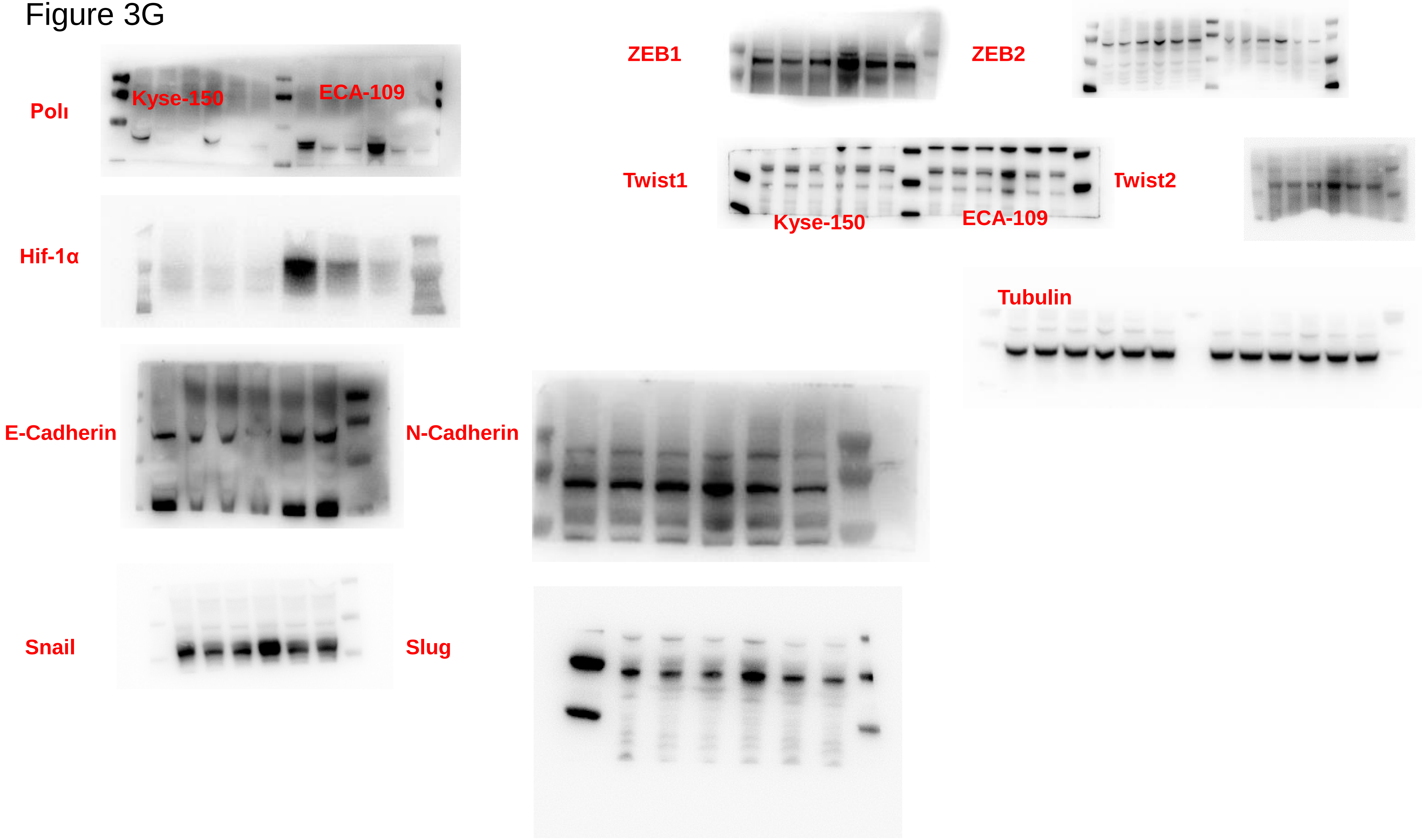
Polι
Kyse-150
ECA-109
Hif-1α
E-Cadherin
N-Cadherin
Snail
Slug
ZEB1
ZEB2
Twist1
Kyse-150
ECA-109
Twist2
Tubulin

Figure 3H

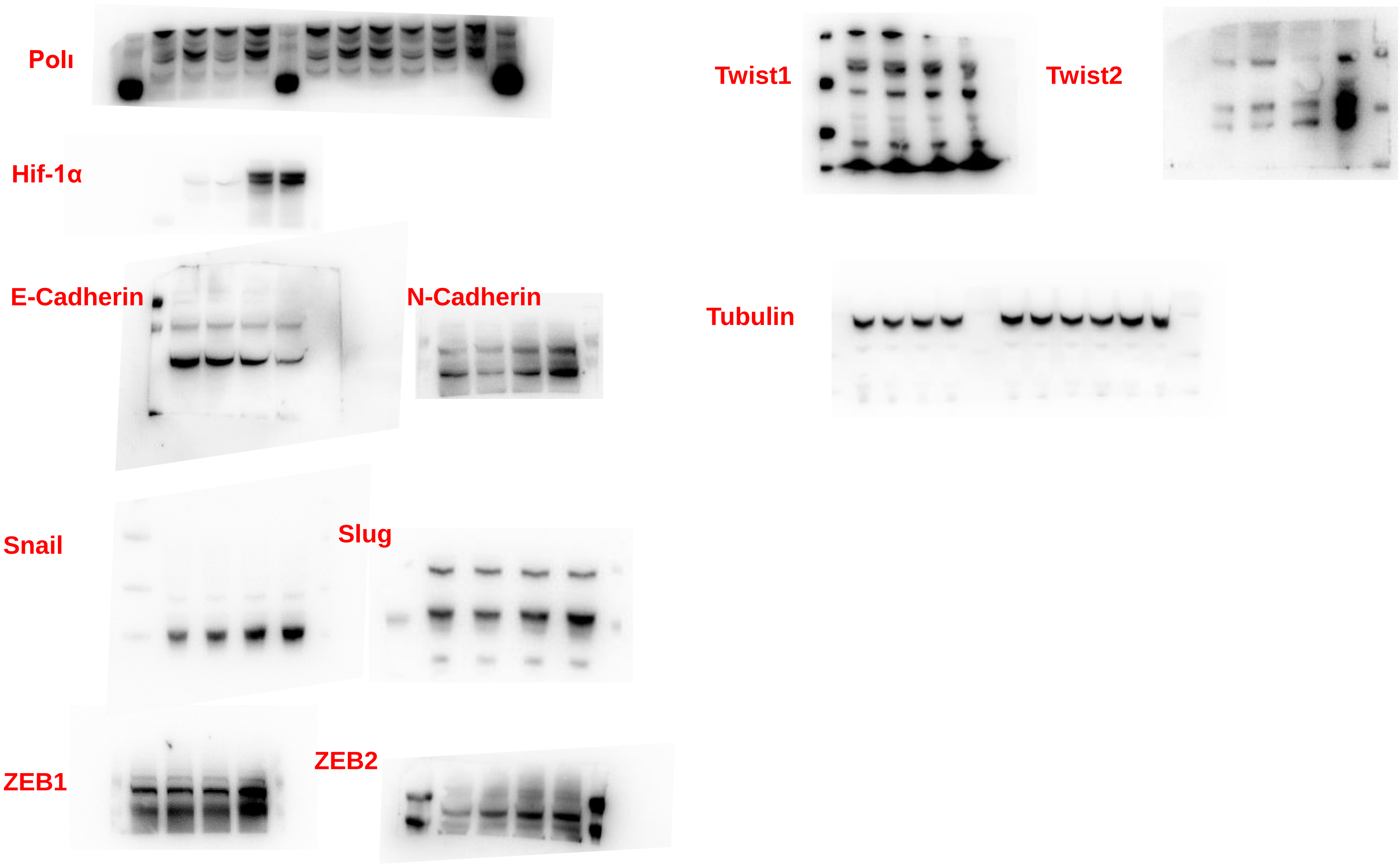

Polι
Hif-1α
E-Cadherin
N-Cadherin
Snail
Slug
ZEB1
ZEB2
Twist1
Twist2
Tubulin

Figure 4

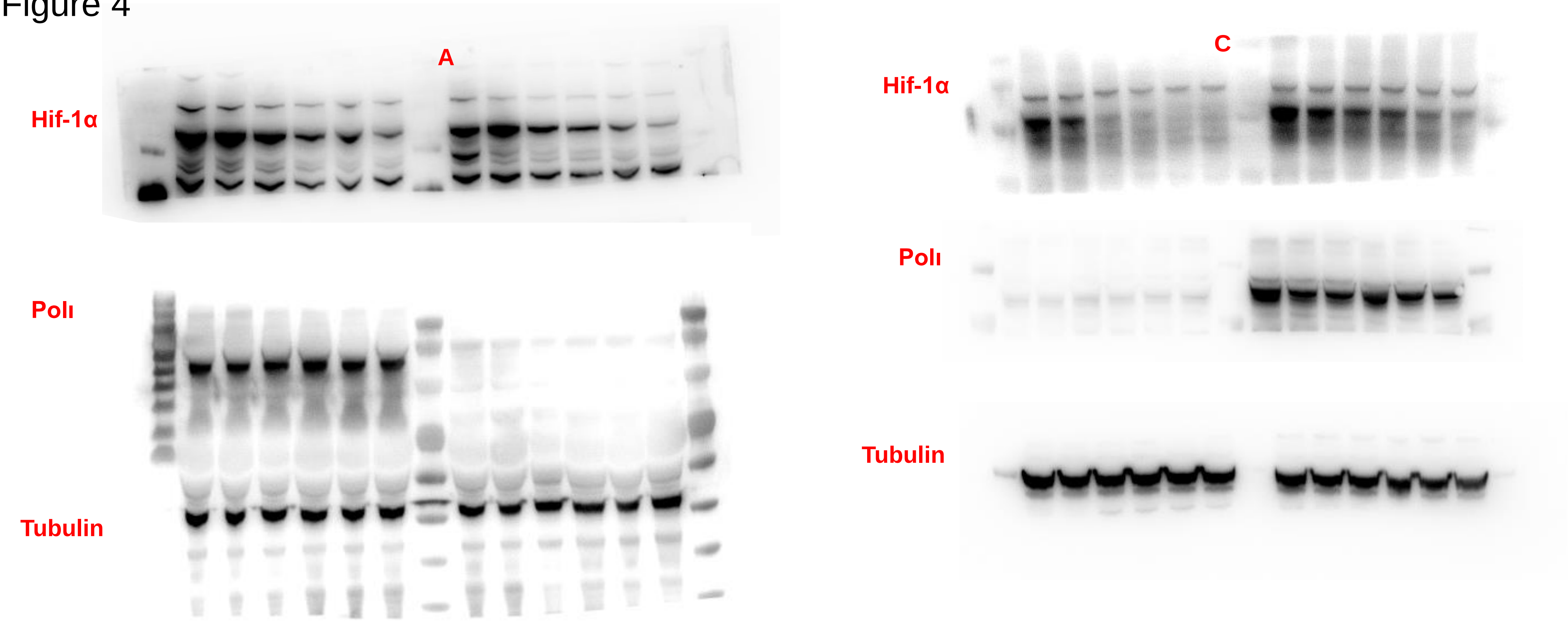

Figure 4

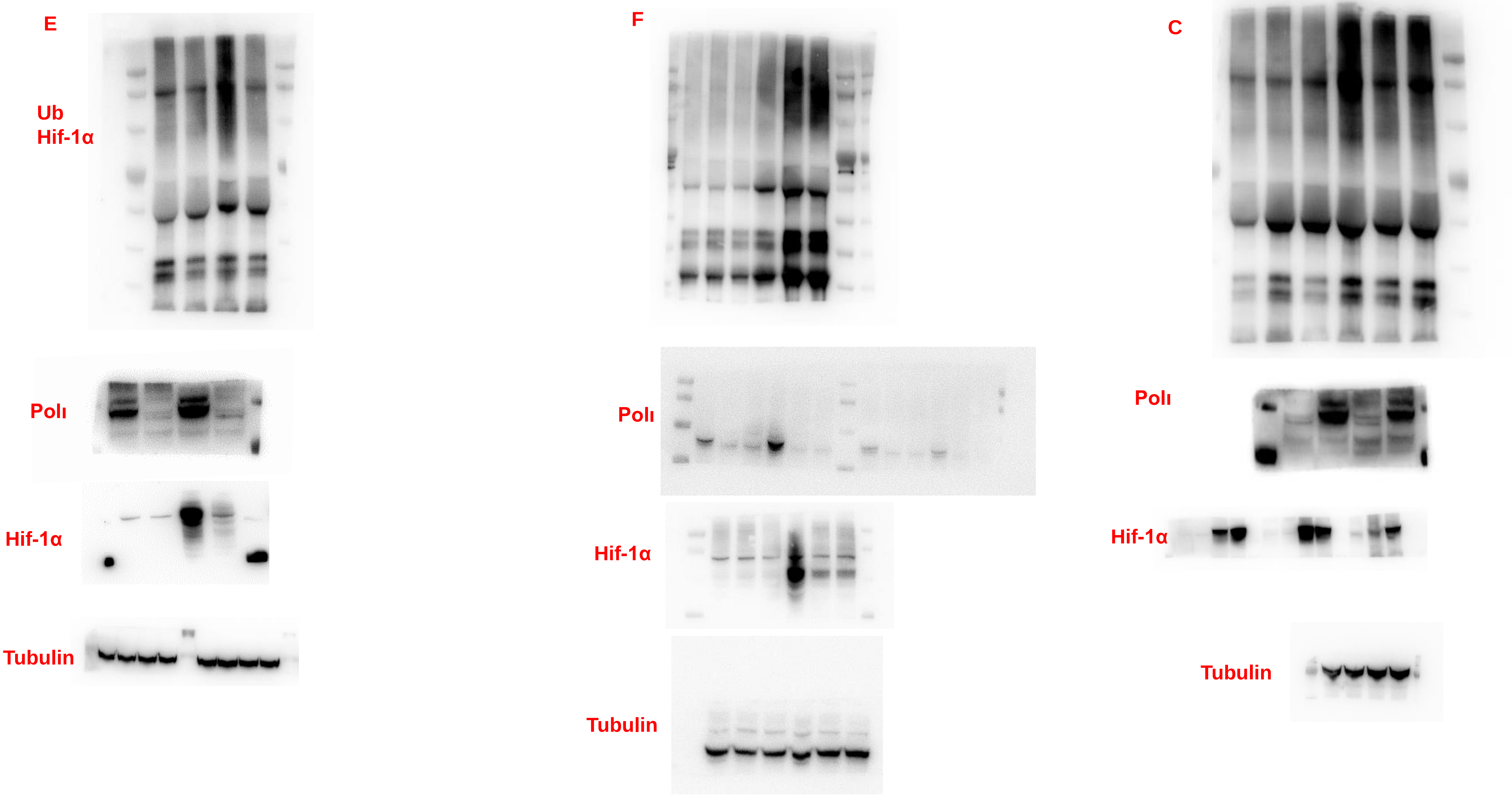
E
Ub
Hif-1α
Polı
Hif-1α
Tubulin
F
Polı
Hif-1α
Tubulin
C
Polı
Hif-1α
Tubulin

Figure 4

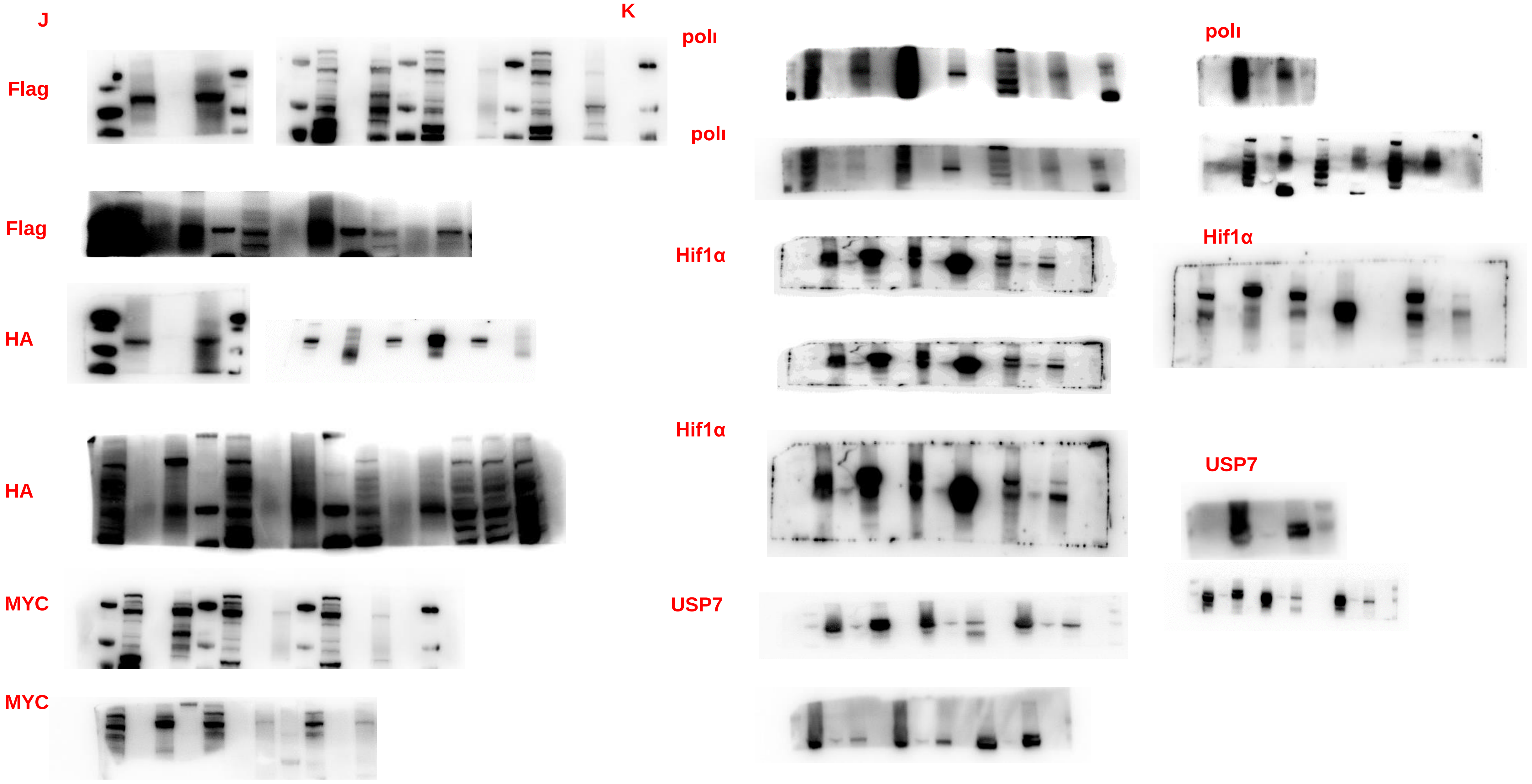

J
K
Flag
Flag
HA
HA
MYC
MYC
polι
polι
polι
Hif1α
Hif1α
Hif1α
USP7
USP7

Figure 4

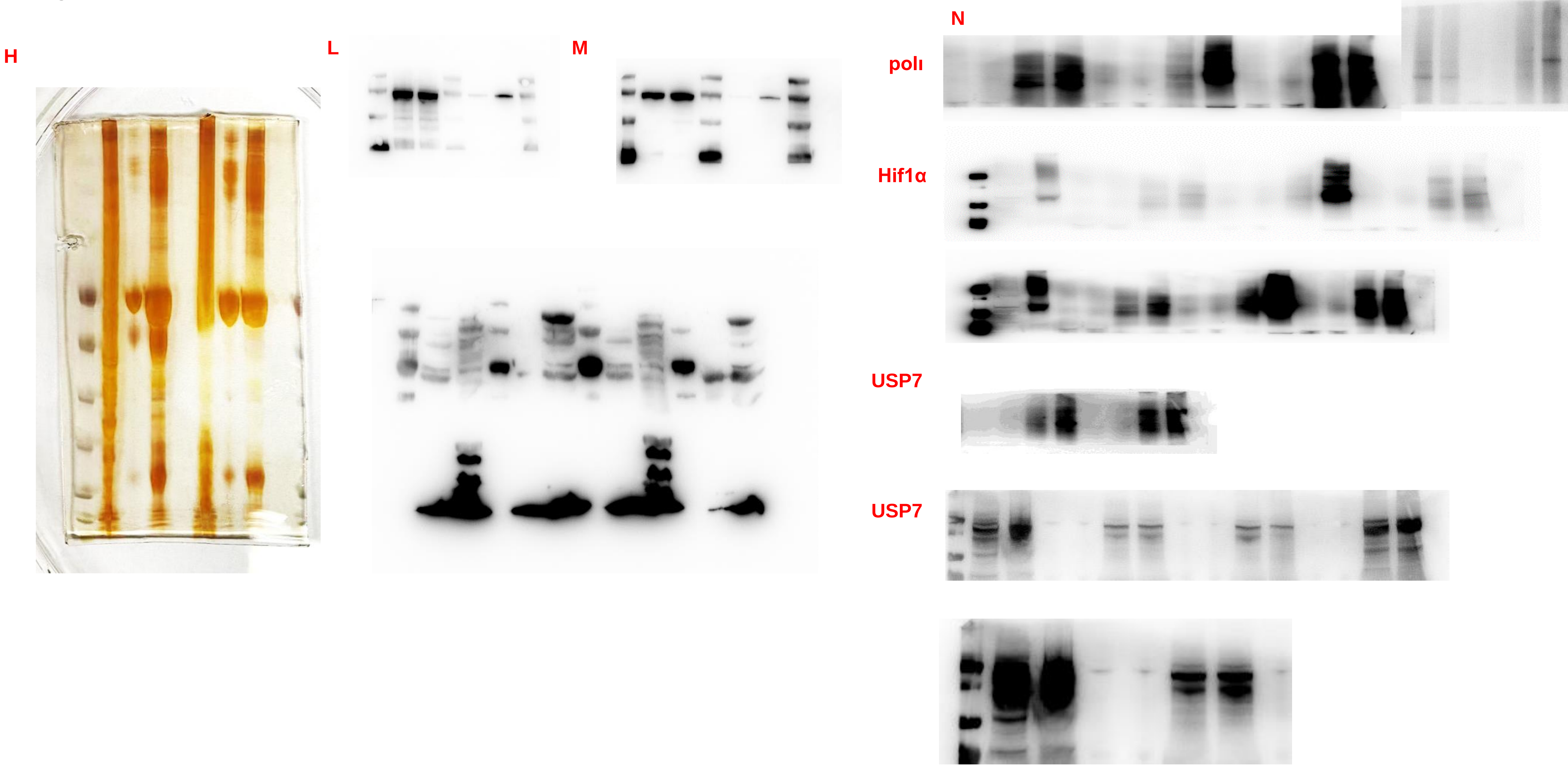

Figure 5

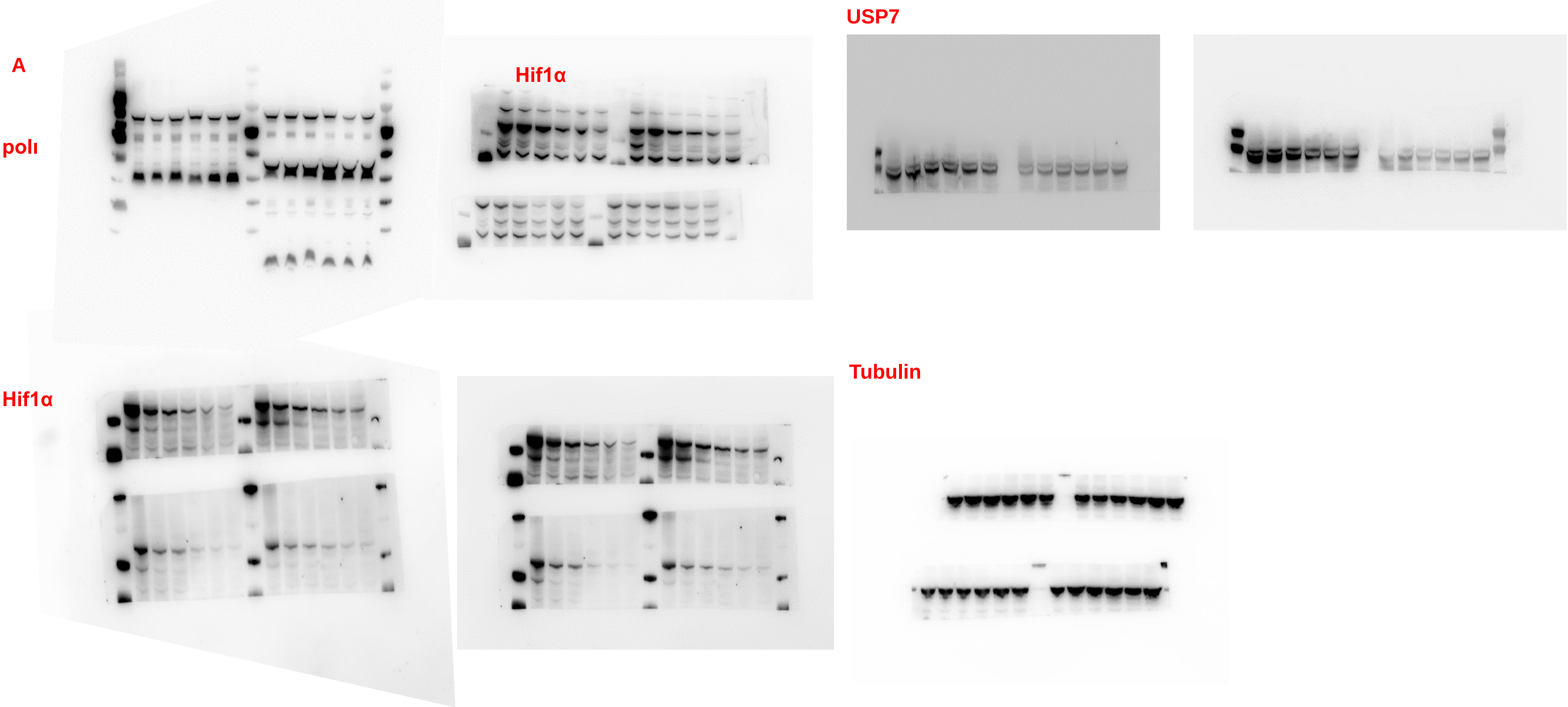

Figure 5

B

polı

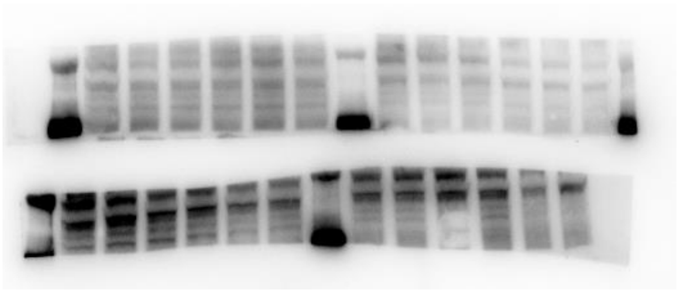

Hif1α

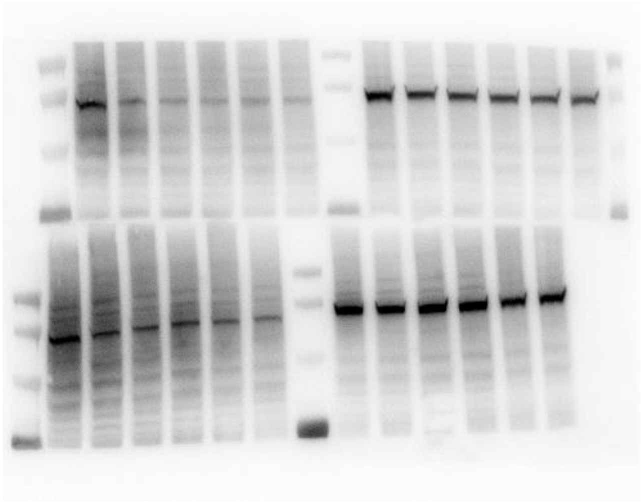

USP7

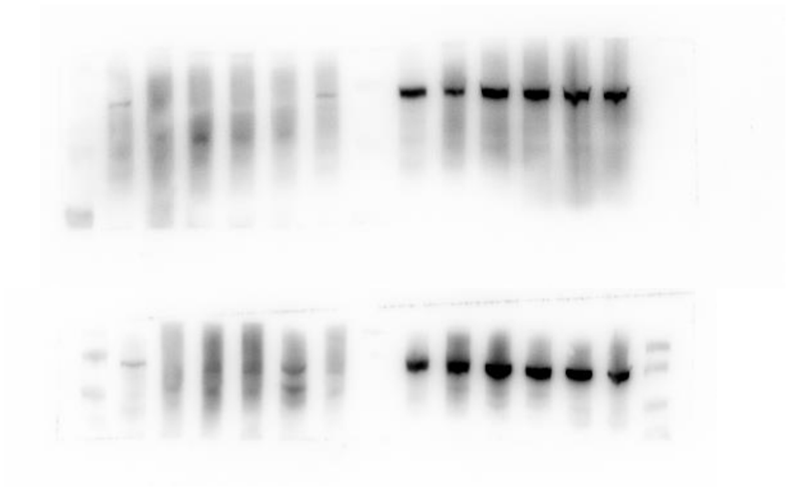

Tubulin

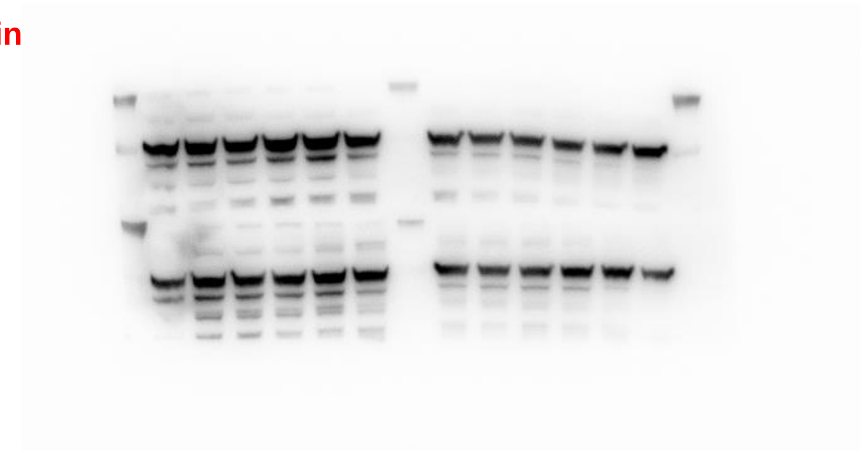

Figure 5

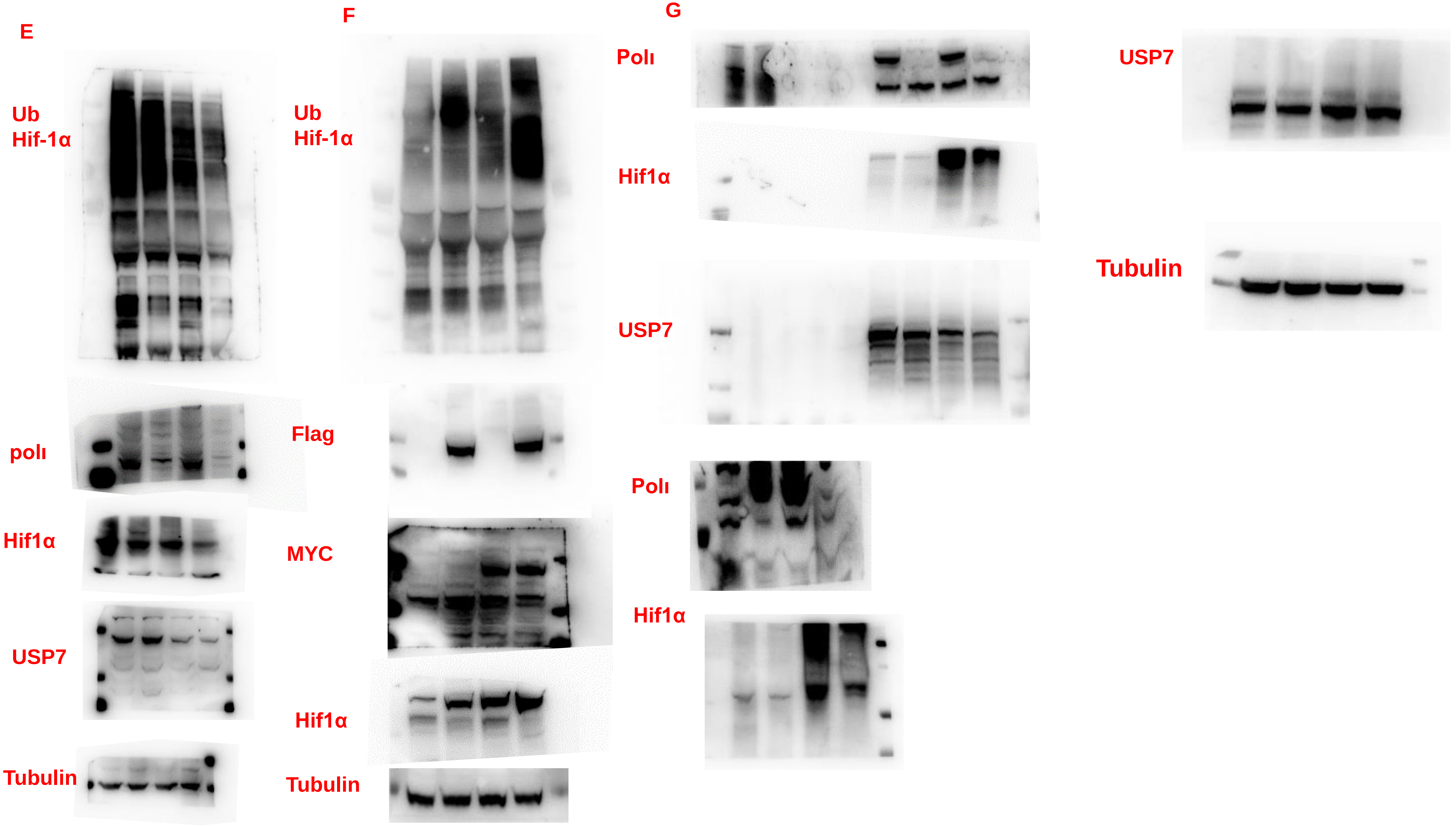
E
Ub
Hif-1α
polı
Hif1α
USP7
Tubulin
F
Ub
Hif-1α
Flag
MYC
Hif1α
Tubulin
G
Polı
Hif1α
USP7
Polı
Hif1α
USP7
Tubulin

Figure 5

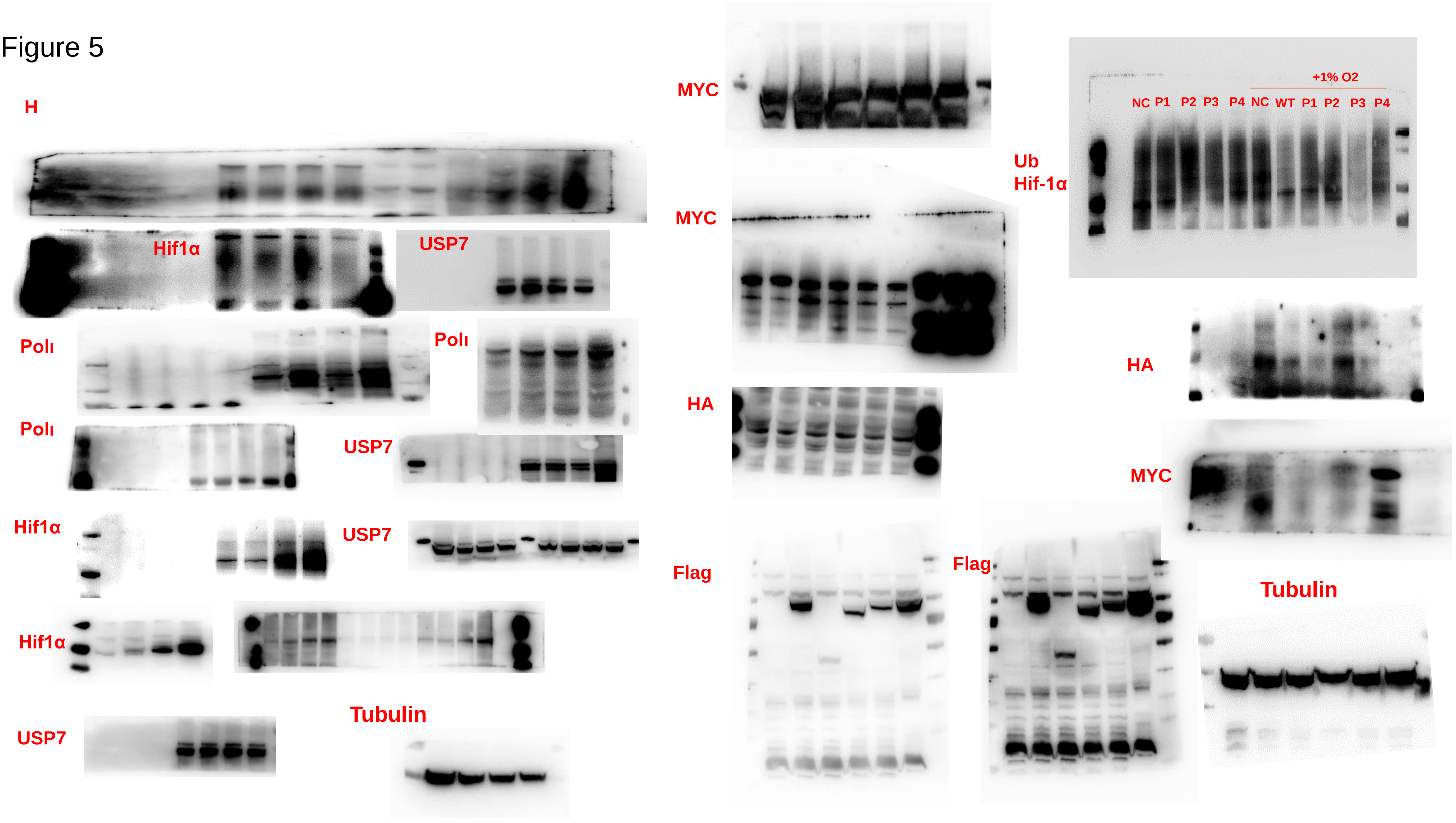

H
Hif1α
USP7
Polı
Polı
Polı
USP7
Hif1α
USP7
Hif1α
USP7
Tubulin
MYC
MYC
HA
Flag
Flag
Ub
Hif-1α
+1% O2
NC P1 P2 P3 P4 NC WT P1 P2 P3 P4
HA
MYC
Tubulin

Figure 5

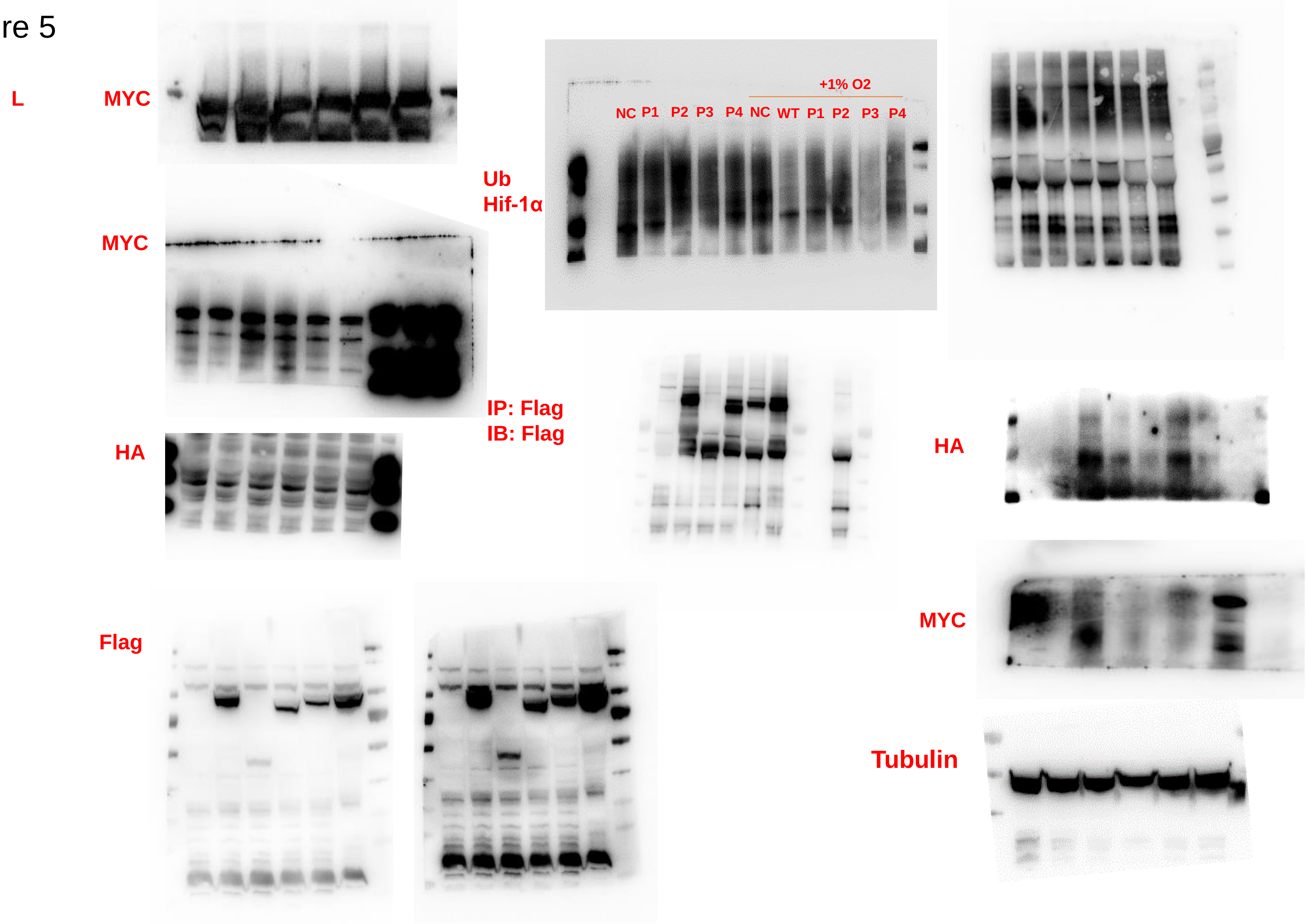
L
MYC
MYC
HA
Flag
Ub
Hif-1α
+1% O2
NC P1 P2 P3 P4 NC WT P1 P2 P3 P4
IP: Flag
IB: Flag
HA
MYC
Tubulin

Figure 6E

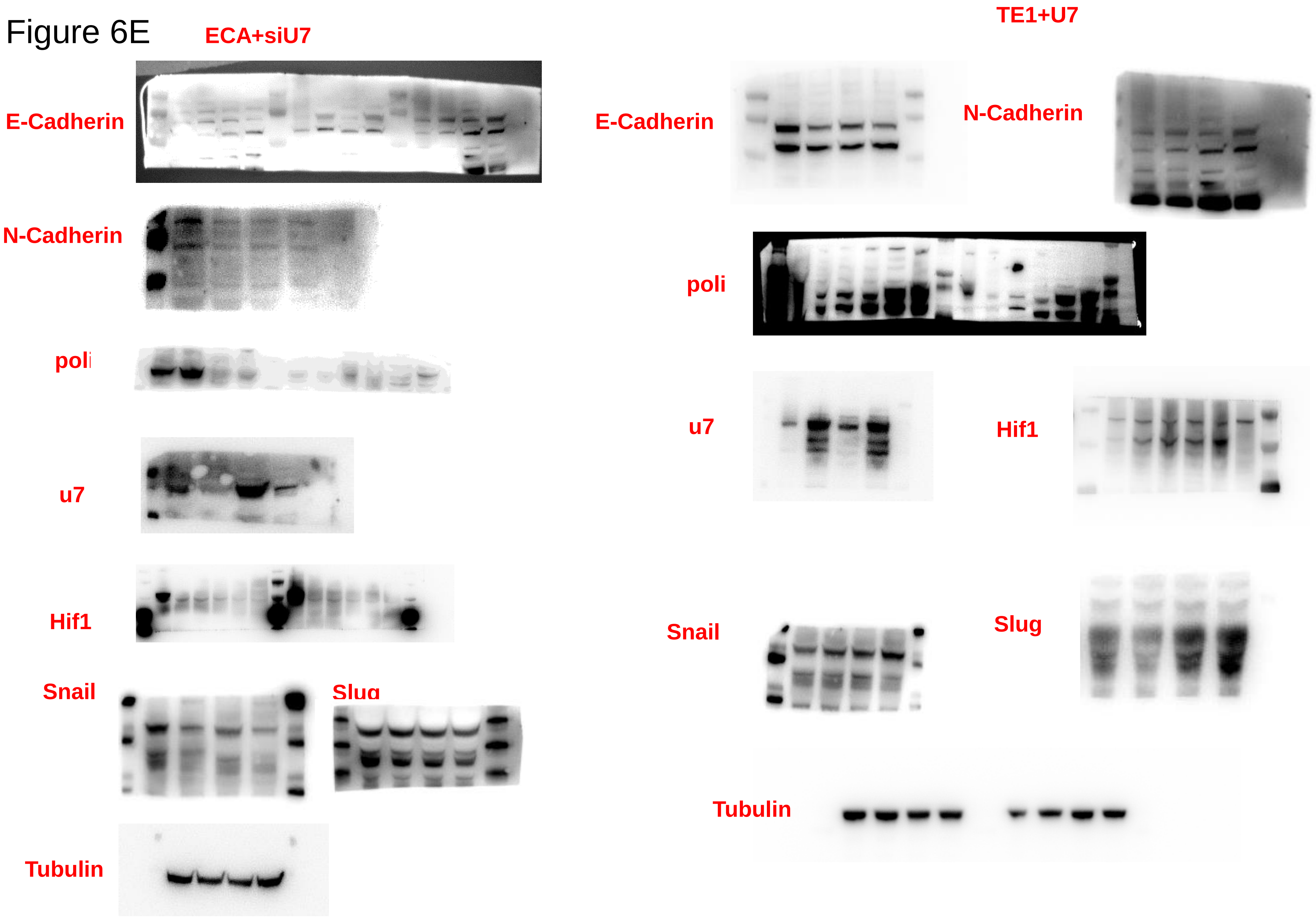
ECA+siU7
E-Cadherin
N-Cadherin
poli
u7
Hif1
Snail
Slug
Tubulin
TE1+U7
E-Cadherin
N-Cadherin
poli
u7
Hif1
Snail
Slug
Tubulin

Figure 6O

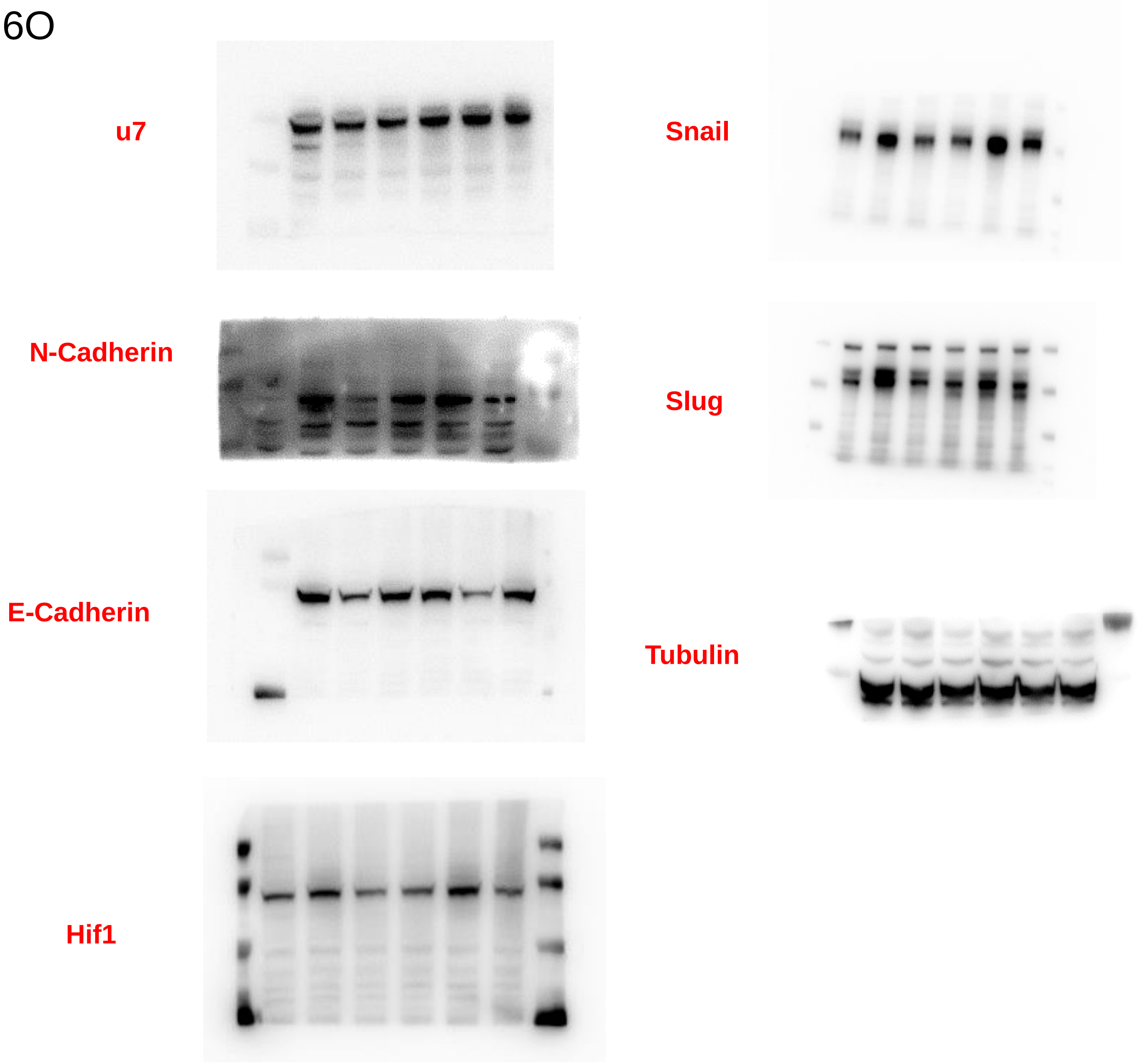

Sipplementary Figure 1

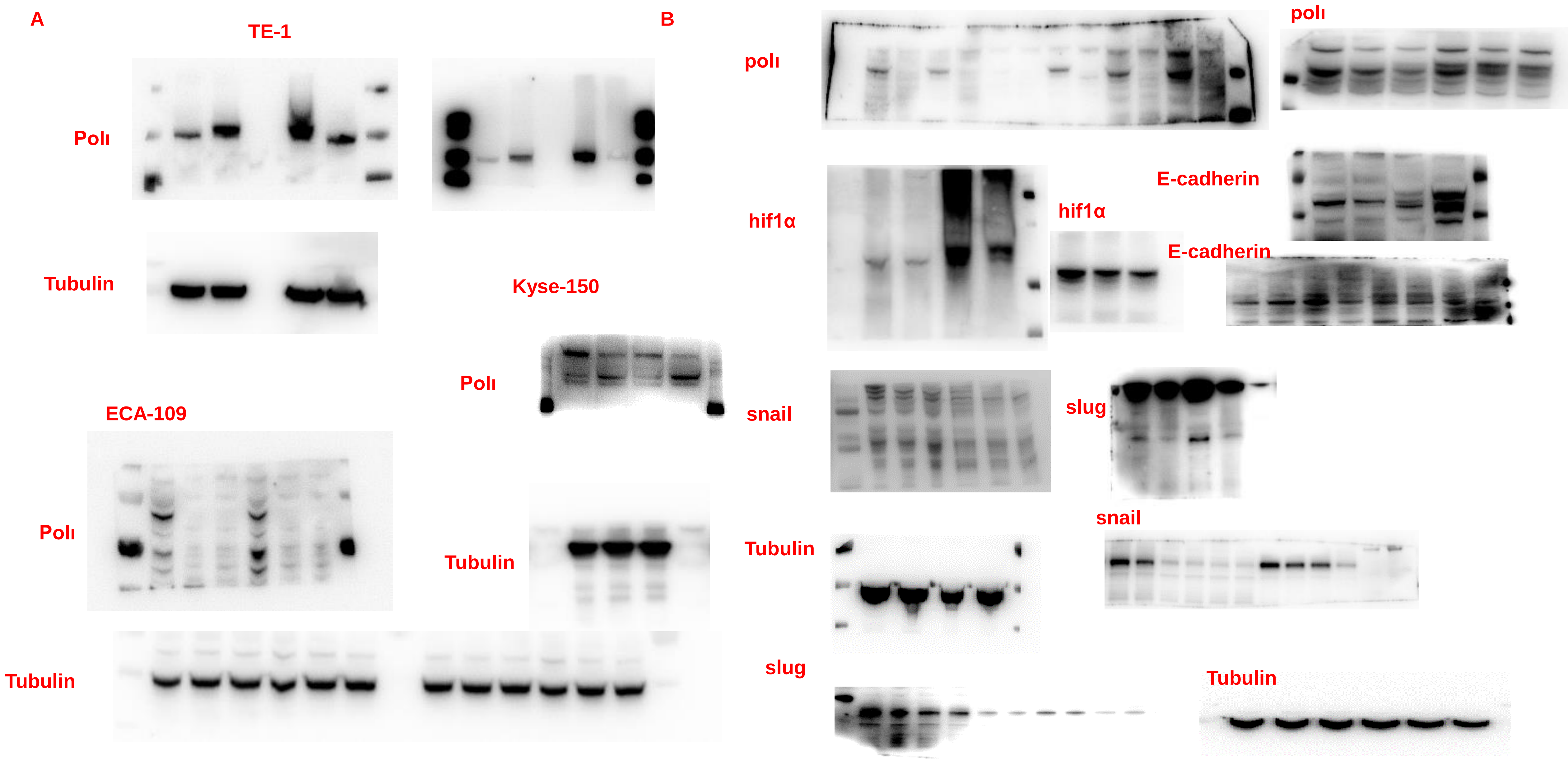

Sipplementary Figure 1

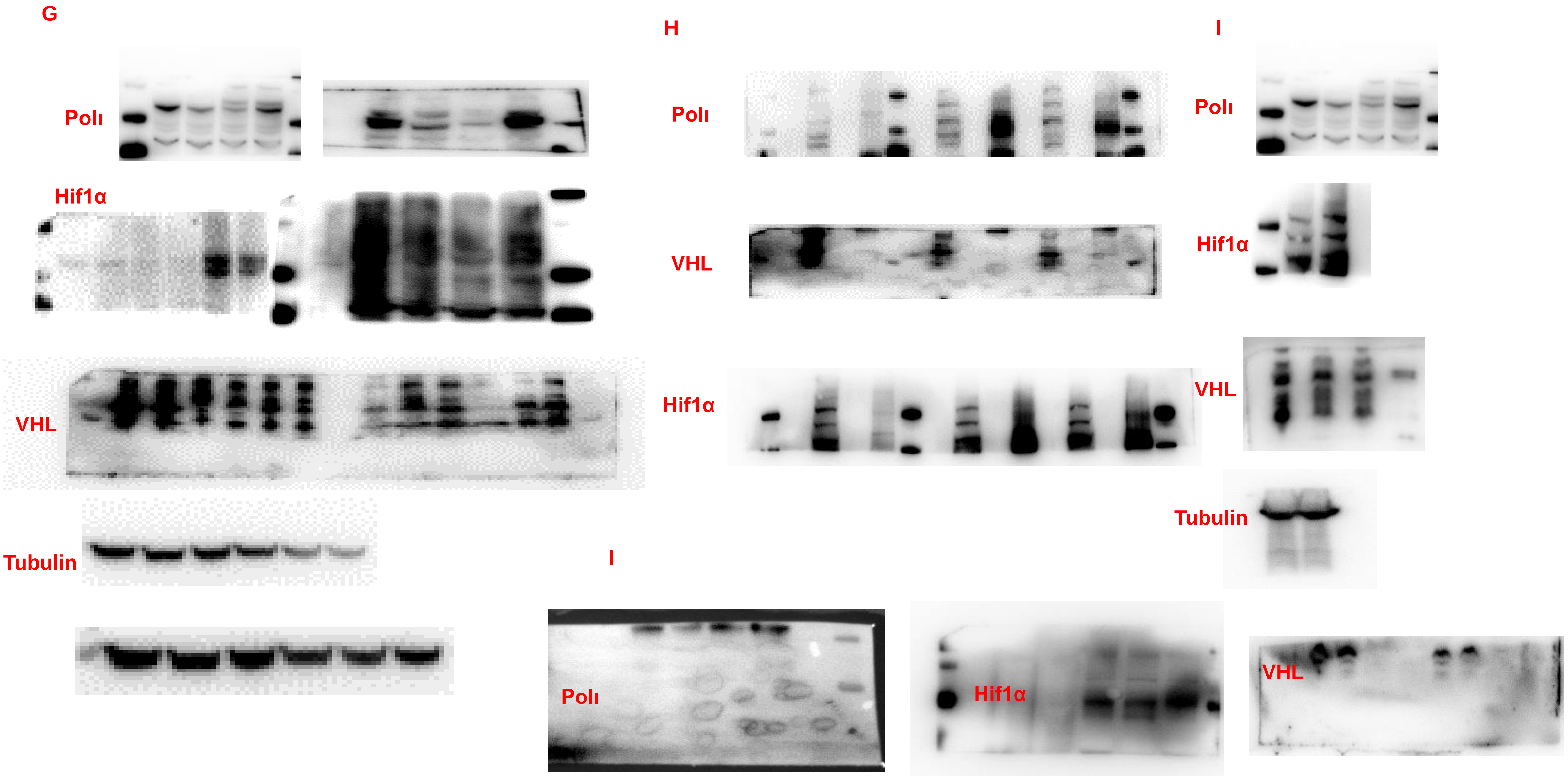

Sipplementary Figure 2

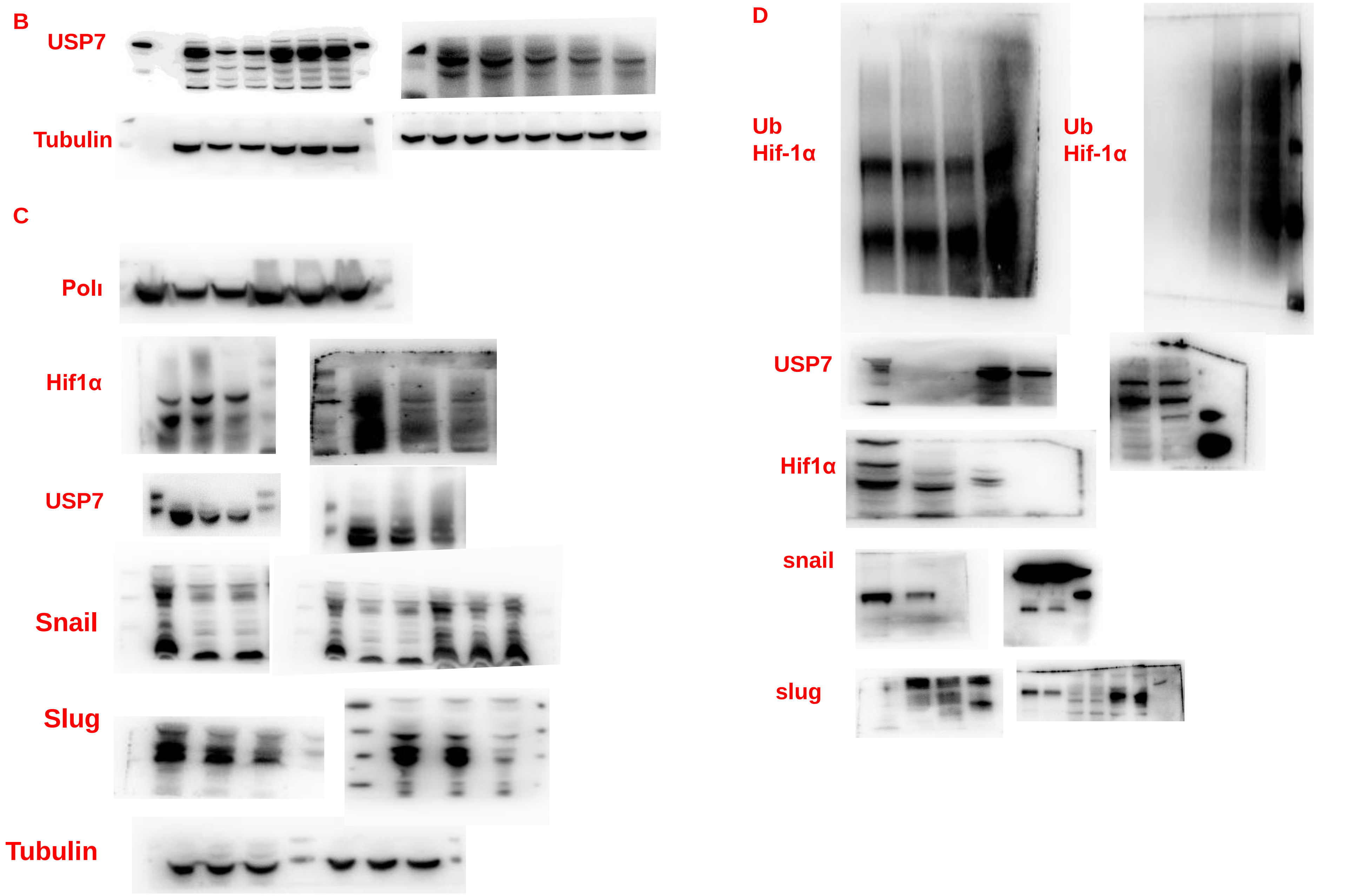

Sipplementary Figure 3

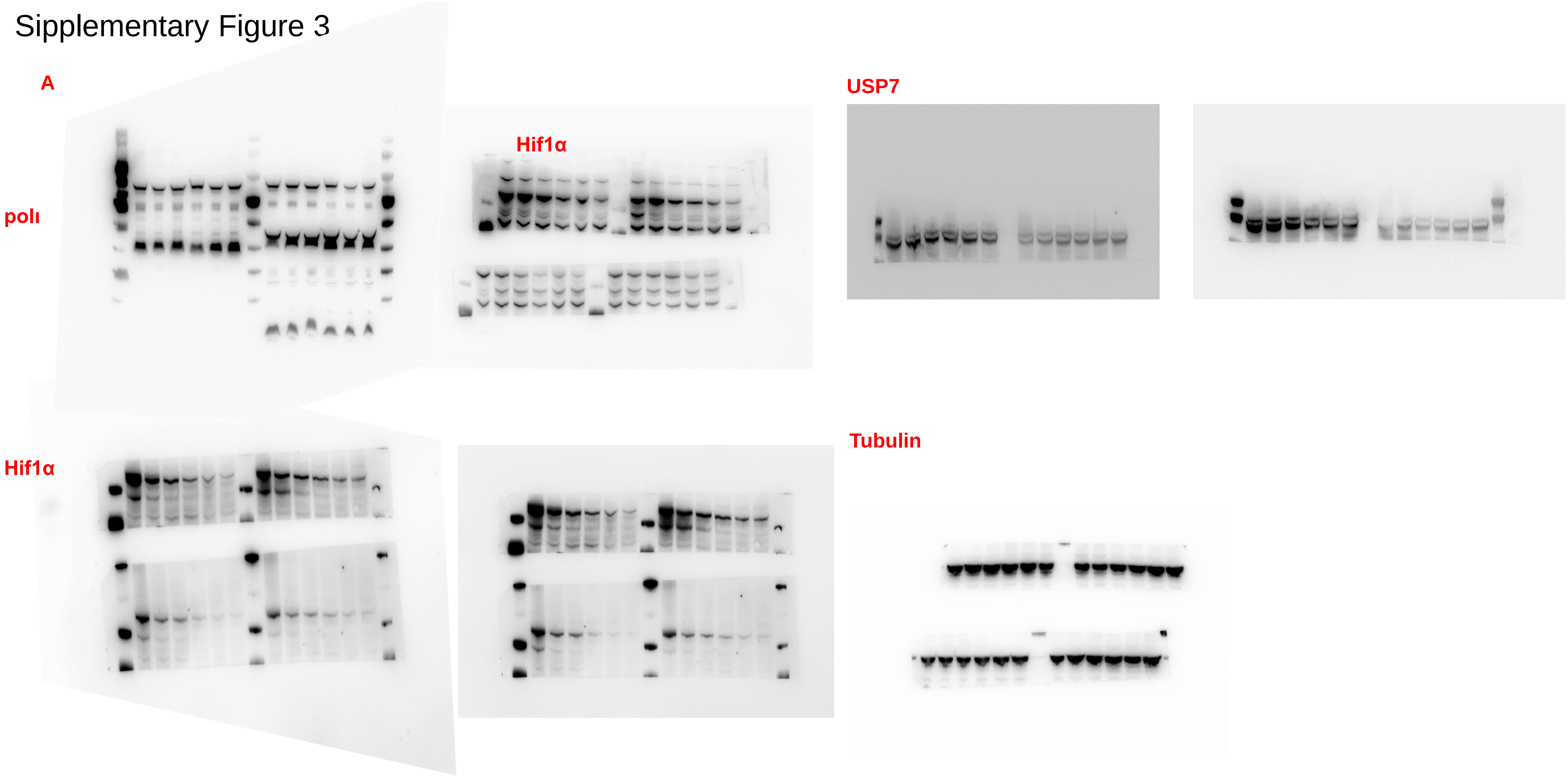

Sipplementary Figure 3

B

polı

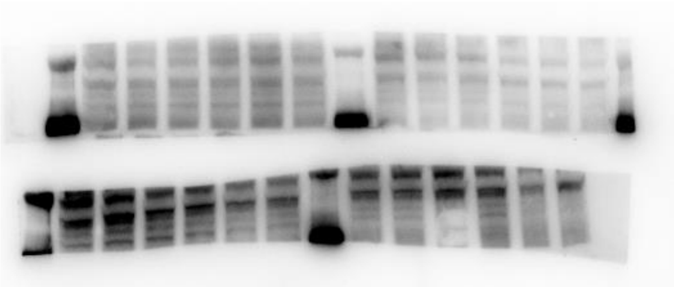

Hif1α

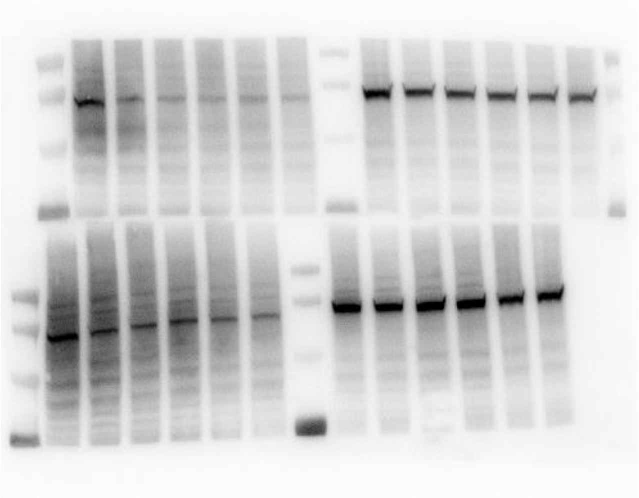

USP7

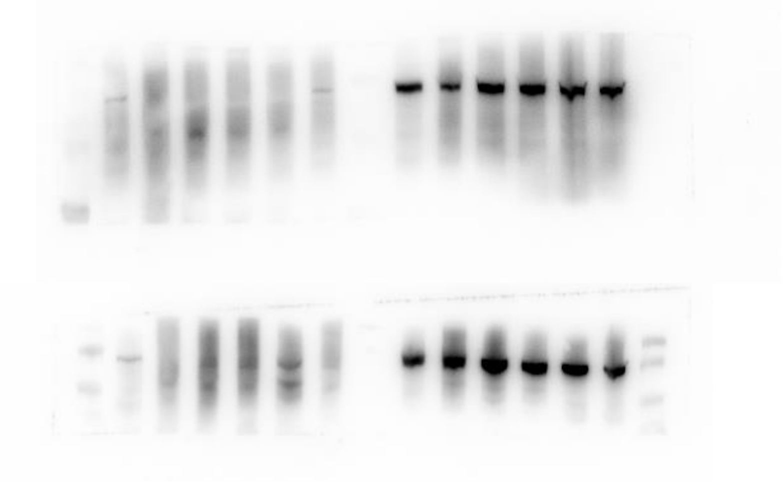

Tubulin

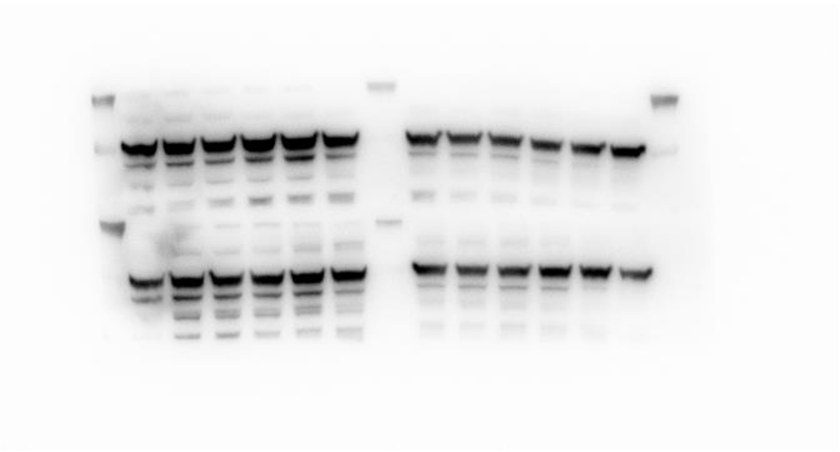

Supplement: Supplementary file 8 — Full and uncropped western blots [file 41419_2024_6552_MOESM8_ESM.pdf]
